# **Peptidoglycan from** ***Bifidobacterium adolescentis*** **enhances IL-10 production in regulatory B cells to alleviate gut inflammation**

**DOI:** 10.1080/19490976.2025.2611603

**Published:** 2026-01-09

**Authors:** Sohyeon Lee, Yoonho Lee, Ho-Su Lee, Jiyoung Yu, Kyunggon Kim, Tae-Young Kim, Su-Hyun Lee, Yuan Qiao, Seungil Kim, Mi-Na Kweon

**Affiliations:** a Mucosal Immunology Laboratory, Department of Convergence Medicine, Asan Medical Center, University of Ulsan College of Medicine, Seoul, Republic of Korea; b Department of Microbiology, Asan Medical Center, University of Ulsan College of Medicine, Seoul, Republic of Korea; c Department of Biochemistry and Molecular Biology, Asan Medical Center, University of Ulsan College of Medicine, Seoul, Republic of Korea; d Department of Medical Informatics and Statistics, Brain Korea 21 project, Asan Medical Center, University of Ulsan College of Medicine, Seoul, Republic of Korea; e Convergence Medicine Research Center, Asan Institute for Life Sciences, Seoul, Republic of Korea; f Digestive Diseases Research Center, Asan Medical Center, University of Ulsan College of Medicine, Seoul, Republic of Korea; g School of Chemistry, Chemical Engineering and Biotechnology, Nanyang Technological University, Singapore, Singapore; h Food Functionality Research Division, Korea Food Research Institute, Wanju, Republic of Korea

**Keywords:** Regulatory B cells, IL-10, gut microbiota, peritoneal cavity cells, peptidoglycan, TLR2

## Abstract

The mechanisms by which gut microbiota modulate host immune responses remain incompletely understood. Here, we screened *Lactobacillus* and *Bifidobacterium* strains isolated from healthy individuals to identify symbionts capable of suppressing gut inflammation. Among them, *Bifidobacterium adolescentis* (Bifi-94) induced IL-10 production in mononuclear cells *in vitro*. Oral administration of Bifi-94 to mice treated with dextran sulfate sodium attenuated weight loss and reduced colonic inflammation scores. In wild-type C57BL/6 mice, Bifi-94 increased IL-10 levels in colonic tissue homogenates without altering the frequency of regulatory T cells. Instead, CD19^+^CD11b^+^ regulatory B (Breg) cells emerged as the primary source of IL-10, with their numbers significantly increasing in the peritoneal cavity (PEC) after treatment. IL-10 secretion by PEC cells was robustly activated by live, heat-killed, and formalin-fixed Bifi-94. Bifi-94-derived peptidoglycan (PG) selectively stimulated IL-10 production in CD19⁺CD11b⁺ Breg cells, and multi-omics analyses showed that Bifi-94 exhibits increased expression of PG biosynthetic enzymes (MurE, MurD, Alr, UppP) relative to the type strain. Mechanistically, Bifi-94-derived PG promoted TLR2-dependent activation of ERK and p38 MAPK signaling in Breg cells. Notably, PG similarly enhanced IL-10 production in CD19^+^ B cells from human colonic tissue. These findings demonstrate that Bifi-94-derived PG promotes IL-10 production in Breg cells via TLR2-mediated signaling, thereby contributing to the attenuation of gut inflammation.

## Introduction

The human gastrointestinal tract harbors a highly diverse and dynamic microbial ecosystem, known as the gut microbiota, which plays a central role in maintaining intestinal homeostasis and regulating host immunity.^1^^,^^2^ Among them, *Lactobacillus* and *Bifidobacterium* species have been recognized for their health-promoting properties.^3^^,^^4^ These symbionts are widely recognized as probiotics that modulate immune responses by interacting with epithelial and immune cells and producing a range of bioactive metabolites.^5^^,^^6^ Such microbial-immune interactions are critical for maintaining immune tolerance and preventing excessive inflammation.

Microbial-derived metabolites and cell wall components profoundly influence host immunity. Short-chain fatty acids (SCFAs), such as butyrate and propionate, promote the differentiation of regulatory T (Treg) cells, which suppress effector T cell responses.^7^ Similarly, tryptophan metabolites such as indole-3-aldehyde and indoleacrylic acid help preserve mucosal immunity and intestinal barrier integrity.^8^^,^^9^ Among these cell wall components, peptidoglycan (PG), a key structural component of bacterial cell walls, has emerged as a potent immunomodulatory factor. For instance, PG derived from *Lactobacillus salivarius* alleviates colitis in an IL-10-dependent manner by promoting CD103⁺ regulatory dendritic cells (DCs) and Treg cells.^10^ Additionally, PG has also been shown to suppress pro-inflammatory cytokine production in lipopolysaccharide (LPS)-stimulated macrophages^11^ and enhance Treg differentiation through *N*-acetylglucosamine (GluNAc), a PG subunit, while also enhancing Treg differentiation and suppressing pro-inflammatory T helper 1 (Th1) and Th17 cell responses.^12^ Muramyl dipeptide (MDP), a NOD2 ligand derived from PG, and DL-endopeptidase-producing *Lactobacillus salivarius* strains have been shown to attenuate dextran sulfate sodium (DSS)-induced colitis via NOD2 signaling.^13^

IL-10 is a central anti-inflammatory cytokine that regulates immune responses and plays a critical role in maintaining the integrity of the intestinal barrier.^14^ Multiple immune cell types, including Foxp3⁺ Treg cells, macrophages, and B cells, are capable of producing IL-10.^15^^,^^16^ Previous studies have demonstrated that the gut microbiota can induce IL-10 production in immune cells, thereby alleviating intestinal inflammation. For example, *Clostridium butyricum* and *Bacteroides fragilis-*derived polysaccharide A (PSA) have been shown to enhance IL-10 production in macrophages and Treg cells, respectively, through Toll-like receptor 2 (TLR2)-dependent signaling, ultimately contributing to the amelioration of colitis.^17^^,^^18^ Additionally, IL-10-producing B cells residing within the colonic lamina propria can be induced by commensal microbiota and play a role in suppressing gut inflammation.^19^ Although numerous studies have shown that symbiotic bacteria can promote IL-10 production and suppress inflammation, the microbial and host mechanisms involved remain poorly understood.

Although Treg cells have been widely recognized for their anti-inflammatory functions, accumulating evidence has revealed a comparable immunosuppressive role for regulatory B (Breg) cells. Breg cells comprise a specialized subset of B cells that suppress immune responses primarily through the production of IL-10.^20^ These IL-10-producing Breg cells dampen inflammation by inhibiting the differentiation of Th1 and Th17 cells, as well as reducing cytokine secretion.^21^^,^^22^ Several phenotypically distinct Breg subsets have been identified, including B220^low/mid^CD19⁺CD11b⁺ cells (B-1 B cells),^23^^,^^24^ CD19⁺TIM1⁺ cells (TIM1⁺ B cells),^25^ and CD19⁺CD5⁺CD1d^hi^ cells (B10 cells).^26^ The protective role of Breg cells has been demonstrated in several murine models of immune-mediated diseases, including colitis, experimental autoimmune encephalomyelitis (EAE), and arthritis.^27^^,^^28^ Despite this growing body of evidence, the molecular mechanisms that govern IL-10 production by B cells remain to be fully elucidated.

In this study, we investigated the immunoregulatory properties of a human-derived strain of *Bifidobacterium adolescentis*, with a particular focus on its ability to modulate immune cell function and promote IL-10 production. Our findings demonstrate that PG derived from *B. adolescentis* significantly enhances IL-10 production in Breg cells in a TLR2-dependent manner. Furthermore, we found that this PG also stimulates IL-10 secretion by B cells isolated from human colonic tissue. These results identify *B. adolescentis* as a potent immunoregulatory symbiont and suggest its therapeutic potential in promoting anti-inflammatory responses in the context of gut inflammation.

## Results

### Anti-inflammatory properties of *B. adolescentis* isolated from human feces

**Figure 1.**
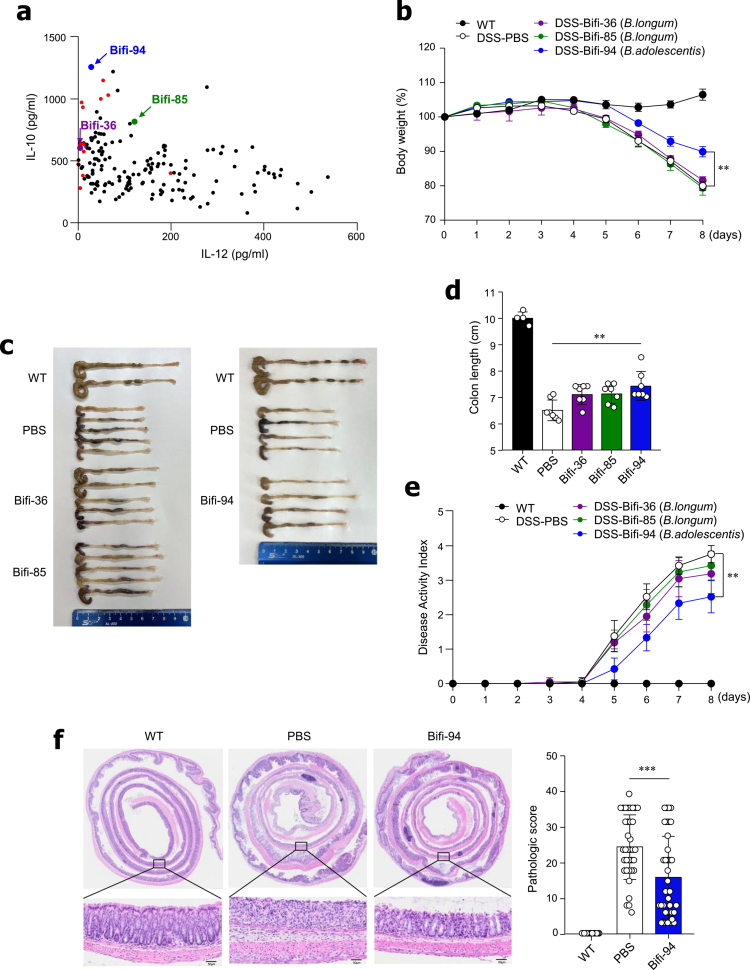
Oral administration of *B. adolescentis* Bifi-94 ameliorates DSS-induced colitis. (a) Scatter plot showing the IL-10/IL-12 ratio from initial bacterial screening. Red dots represent the 15 tested isolates, while Bifi-36, Bifi-85, and Bifi-94 are highlighted as purple, green, and blue dots, respectively. (b) Changes in body weight over time (*n* = 7). (c) Representative colon images (*n* = 4−9) and (d) quantification of colon length (*n* = 5−9). (e) Disease activity index (*n* = 7). (f) Representative H&E-stained colon sections and corresponding histological scores (*n* = 9). Data are presented as mean ± SD. Statistical analysis was performed using one-way ANOVA followed by Tukey’s post-hoc test or two-way ANOVA followed by Bonferroni’s post-hoc test, as appropriate. **p < 0.01 and ***p < 0.001 were considered statistically significant.

To identify symbiotic bacteria with potent anti-inflammatory properties, we isolated multiple *Lactobacillus* and *Bifidobacterium* strains from the feces of healthy human donors. These isolates were screened using a splenocyte–bacterium co-culture system to identify strains that preferentially elicit anti-inflammatory responses. We focused on those that robustly promote IL-10 production while minimizing IL-12 secretion, as IL-12 derived from DCs drives inflammatory Th1 differentiation, whereas IL-10 acts as a key regulatory cytokine that suppresses excessive inflammation.^14^^,^^29^ Approximately 15 strains, including Bifi-36, Bifi-85, and Bifi-94, were identified as strong IL-10 inducers with minimal IL-12 production. These strains were selected for subsequent evaluation of their immunoregulatory activities (Figure 1a).

### Oral administration of *B. adolescentis* Bifi-94 ameliorates DSS-induced colitis

To evaluate the *in vivo* anti-inflammatory potential of the selected bacterial strains, we employed a dextran sulfate sodium (DSS)-induced colitis model in mice. Mice were orally administered PBS or candidate strains daily for two weeks, followed by exposure to 3.5% DSS in drinking water for 7 days to induce acute colitis. Among the tested strains, oral administration of *B. adolescentis* Bifi-94 significantly attenuated body weight loss compared with the PBS, Bifi-36, and Bifi-85 groups (Figure 1b). Bifi-94 treatment also mitigated DSS-induced colon shortening (Figure 1c and 1d), and the disease activity index (DAI) was markedly lower in Bifi-94-treated mice (Figure 1e). Furthermore, histological analysis revealed significantly lower inflammation scores in the colonic tissues from Bifi-94-treated mice relative to PBS-treated controls (Figure 1f). The remaining 12 candidate strains did not confer significant protective effects in this model (data not shown). Collectively, these findings suggest that the newly isolated *B. adolescentis* Bifi-94 is a promising symbiont with potent anti-inflammatory effects.

### Bifi-94 induces IL-10 production independently of CD4⁺Foxp3⁺ T cells

**Figure 2.**
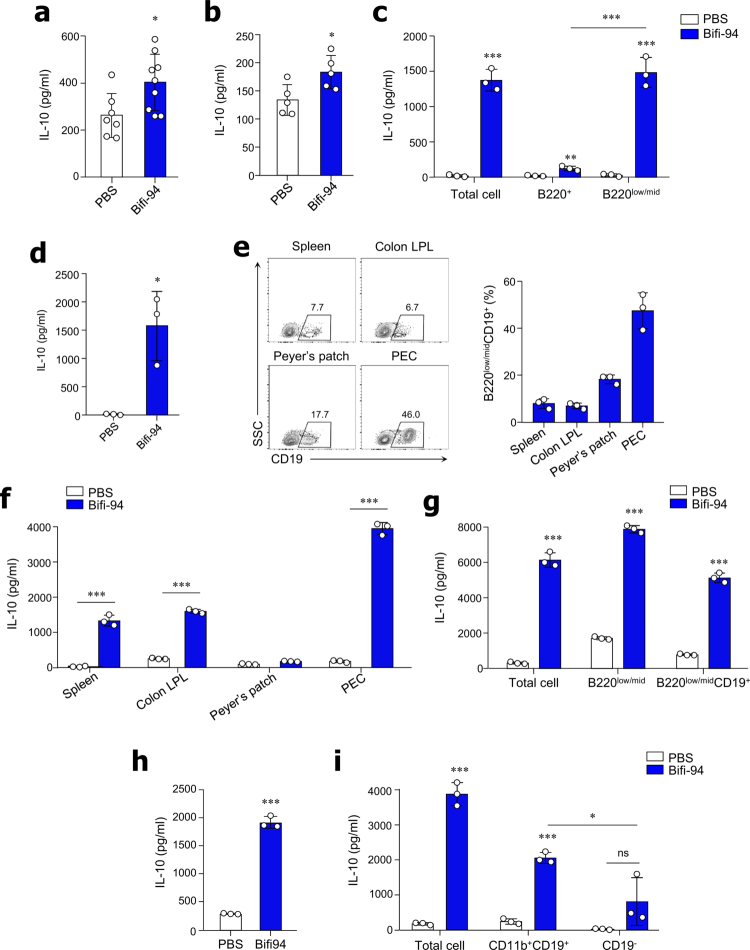
*B. adolescentis* Bifi-94 induces IL-10 production in Breg cells. (a–b) B6 mice were orally administered *B. adolescentis* Bifi-94 for 2 weeks. IL-10 levels were measured in (a) colon tissues (*n* = 7–9) and (b) spleen homogenates (*n* = 5). (c–d) Splenocytes from B6 mice were sorted and co-cultured with live Bifi-94 for 72 hours. IL-10 levels in culture supernatants were quantified by ELISA (*n* = 3). Data were pooled from ≥3 independent experiments. IL-10 production was analyzed in (**c**) B220⁺ and B220^low/mid^ cells, and (d) B220^low/mid^CD19⁺ cells. (e) Representative flow cytometry plots showing B220^low/mid^CD19⁺ Breg cells in the spleen, colon, Peyer’s patches, and PEC (*n* = 3). (f) IL-10 production was assessed following co-culture of tissue-derived mononuclear cells with Bifi-94 (*n* = 3). (g–i) PEC-derived mononuclear cells from B6 mice were sorted and co-cultured with Bifi-94 for 72 hours, and IL-10 levels in the culture supernatant were measured by ELISA (*n* = 3). Data were combined from ≥3 independent experiments. IL-10 production was analyzed in (g) B220^low/mid^, B220^low/mid^CD19⁺ cells, (h) B220^low/mid^ CD11b⁺CD19⁺ cells, and (i) CD11b⁺CD19⁺ and CD19^–^ cell populations. Data are presented as mean ± SD. Statistical analysis was performed using a two-tailed paired Student’s *t*-test or one-way ANOVA followed by Tukey’s post-hoc test, as appropriate. *p < 0.05, **p < 0.01 and ***p < 0.001 were considered statistically significant.

To determine which immune cell populations mediate the anti-inflammatory effects of Bifi-94, we orally administered Bifi-94 to wild-type C57BL/6 (B6) mice for two weeks. As expected, Bifi-94 treatment significantly increased IL-10 levels in tissue homogenates from both the colon and spleen compared with PBS-treated controls (Figure 2a and 2b), whereas IL-12 levels remained unchanged (Figure S1a and S1b). Because CD4^+^Foxp3^+^ Treg cells are a major IL-10-producing population in the intestinal mucosa,^30^^,^^31^ we next examined whether Bifi-94 influenced their abundance. However, the frequency of CD4⁺Foxp3⁺ Treg cells in these tissues remained unchanged (Figure S1c and S1d). To further assess whether Bifi-94 affects Treg cell differentiation, naïve CD4⁺ T cells were isolated from the spleens of wild-type B6 mice and co-cultured with live Bifi-94 under Treg-inducing conditions. Bifi-94 had no effect on CD4⁺Foxp3⁺ T cell differentiation or IL-10 production in this setting (Figure S1e and S1f). In addition, sorted CD3⁺CD4⁺ and CD3⁺CD4⁻ splenic populations did not produce IL-10 upon Bifi-94 stimulation (Figure S1g). Collectively, these findings indicate that Bifi-94 enhances IL-10 production through a mechanism independent of CD4⁺Foxp3⁺ T cells.

### *B. adolescentis* Bifi-94 induces IL-10 production in Breg cells

Given that B cells are also capable of producing IL-10,^32^^,^^33^ we next sorted B220^hi^ and B220^low/mid^ cell populations and co-cultured them with Bifi-94 *in vitro*. Notably, IL-10 was predominantly secreted by the B220^low/mid^ subset (Figure 2c). Previous studies have identified B-1 B cells—characterized by B220^low/mid^, CD19⁺, and CD11b⁺ expression—as potent IL-10 producers.^34^ We therefore sorted B220^low/mid^CD19⁺ cells from the spleen and cultured them with Bifi-94, confirming that these Breg cells robustly produced IL-10 (Figure 2d). These results demonstrate that Bifi-94 selectively induces IL-10 production in B220^low/mid^CD19⁺ Breg cells.

Because Breg cells are relatively rare in the spleen, we next isolated mononuclear cells from the spleen, colon, Peyer’s patches, and peritoneal cavity (PEC) to identify tissues enriched in this population. B220^low/mid^CD19⁺ Breg cells were most abundant in the PEC (Figure 2e), and PEC-derived cells exhibited the highest levels of IL-10 production in response to Bifi-94 stimulation (Figure 2f). IL-10 production by PEC cells increased in a dose-dependent manner when co-cultured with Bifi-94 at cell-to-bacteria ratios of 1:1, 1:5, and 1:10 (Figure S1h). When we sorted B220^low/mid^ and B220^low/mid^CD19⁺ cells from the PEC and stimulated them with Bifi-94, both populations produced IL-10 (Figure 2g). Since Breg cells are further characterized by expression of CD11b, we isolated CD11b⁺CD19⁺ and B220^low/mid^CD11b⁺CD19⁺ subsets and confirmed that both produced IL-10 upon stimulation with Bifi-94 (Figure 2h and 2i).

To explore the effect of Bifi-94 on other Breg cell subtypes, we co-cultured Bifi-94 with mononuclear cells from the spleen, colon, and PEC. In splenocytes and PEC cells, Bifi-94 increased the frequency of TIM1^+^ B cells (Figure S2a and S2b), whereas the frequency of CD19^+^CD5^+^ B cells remained unchanged (Figure S2c and S2d). In contrast, colon-derived mononuclear cells exhibited an increase in CD19^+^CD5^+^ B cells without changes in TIM1^+^ B cells (Figure S2e and S2f). Collectively, these findings indicate that Bifi-94 selectively promotes IL-10 production in CD11b^+^CD19^+^ Breg cells and can modulate multiple Breg subsets across different tissue compartments.

### Oral administration of *B. adolescentis* Bifi-94 enhances IL-10 production by Breg cells

**Figure 3.**
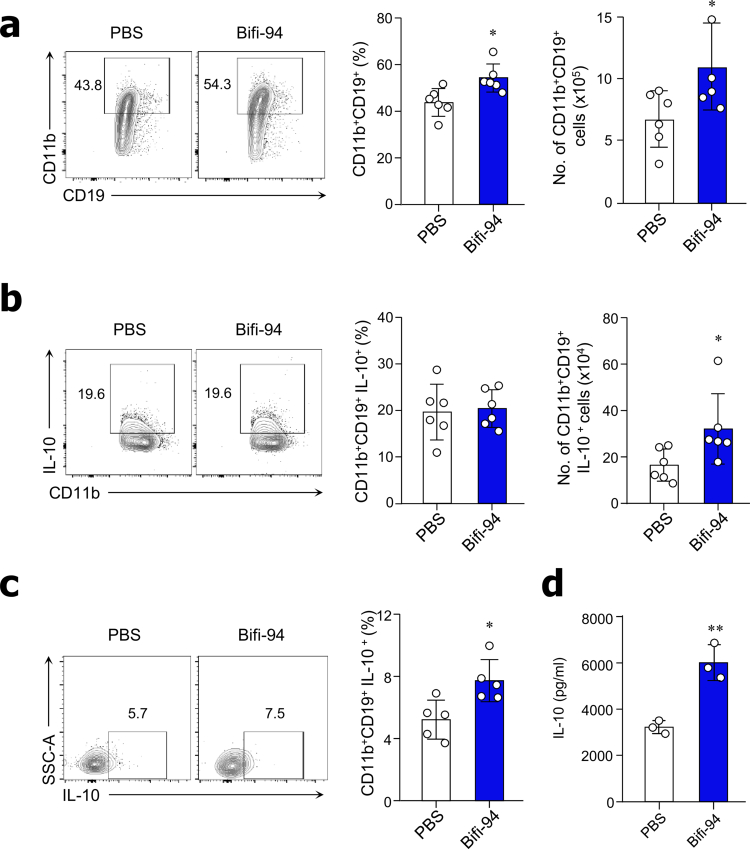
Oral administration of *B. adolescentis* Bifi-94 enhances IL-10 production by Breg cells. B6 mice were orally administered *B. adolescentis* Bifi-94 daily for 4 weeks. (a–c) Breg cell populations and IL-10 expression were analyzed by flow cytometry (*n* = 5–6). (a) Frequency and absolute number of CD11b⁺CD19⁺ Breg cells in the PEC. (b–c) Frequency and number of IL-10^+^ cells among CD11b^+^CD19^+^ Breg cells in the PEC (b) and spleen (c). (d) PEC-derived mononuclear cells were isolated and re-stimulated with Bifi-94 for 72 hours, and IL-10 levels in the culture supernatants were quantified by ELISA (*n* = 3). Data are presented as mean ± SD. Statistical analysis were performed using a two-tailed paired Student’s *t*-test. *p < 0.05, **p < 0.01 and ***p < 0.001 were considered statistically significant.

To determine whether Bifi-94 promotes IL-10 production by Breg cells *in vivo*, wild-type B6 mice were orally administered Bifi-94 daily for 4 weeks. Compared with PBS-treated controls, Bifi-94-treated mice exhibited a significant increase in both the frequency and absolute number of CD11b⁺CD19⁺ Breg cells in the PEC (Figure 3a). Notably, the number of IL-10-producing Breg cells in the PEC was significantly higher in Bifi-94-fed mice than in controls (Figure 3b). Similarly, the frequency of IL-10-producing Breg cells in the spleen was also increased in Bifi-94-treated mice (Figure 3c). To further assess IL-10 production following *in vivo* exposure, mononuclear cells isolated from the PEC of Bifi-94- or PBS-fed mice and restimulated *in vitro* with Bifi-94 for 72 hours. PEC cells from Bifi-94-treated mice exhibited significantly greater IL-10 secretion upon re-stimulation compared with those from PBS-treated mice (Figure 3d). Together, these findings demonstrate that oral administration of Bifi-94 enhances both the abundance and IL-10-producing capacity of CD11b⁺CD19⁺ Breg cells in systemic (spleen) and mucosal (PEC) immune compartments *in vivo.*

### *B. adolescentis* Bifi-94-derived PG enhanced IL-10 production by Breg cells

**Figure 4.**
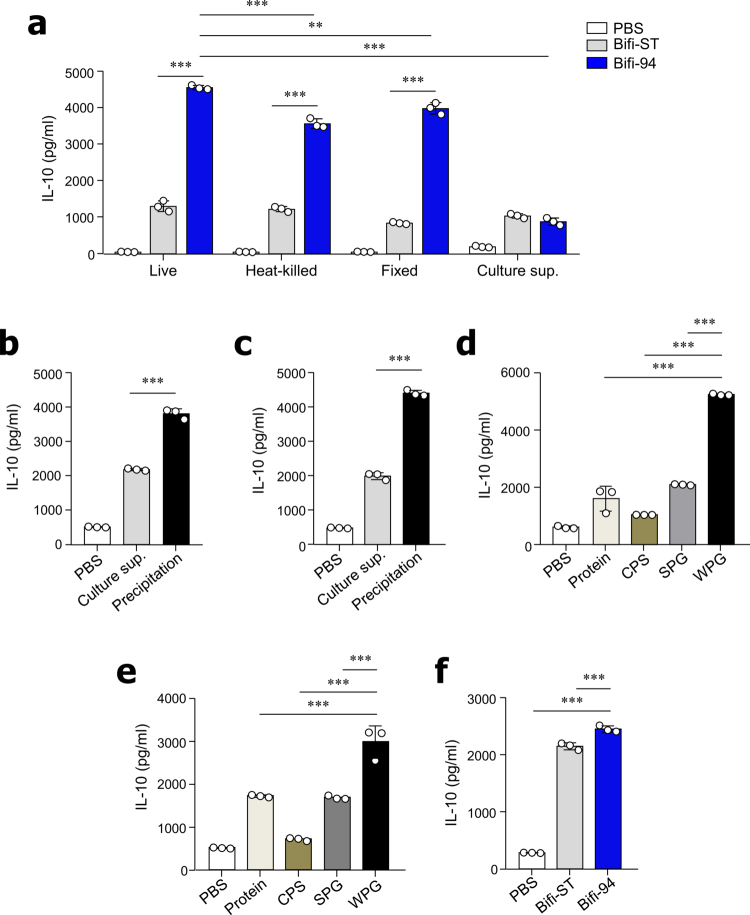
*B. adolescentis* Bifi-94-derived PG enhances IL-10 production by Breg cells. (a–e) PEC cells from B6 mice were incubated with Bifi-94 or its components for 72 hours, and IL-10 levels in the culture supernatants were measured by ELISA. (a) IL-10 production in response to live, heat-killed, or fixed Bifi-94, and its culture supernatant (*n* = 3). (b–c) IL-10 levels in (b) total PEC cells (*n* = 3) and (**c**) sorted CD11b⁺CD19⁺ Breg cells stimulated with the culture supernatant or precipitate of Bifi-94 (*n* = 3). Data were combined from ≥3 independent experiments. (d–e) IL-10 secretion from (d) total PEC cells (*n* = 3) and (e) sorted CD11b⁺CD19⁺ B-1 B cells co-cultured with 10 µg/mL of Bifi-94–derived protein, CPS, SPG, or WPG (*n* = 3). Data were combined from ≥3 independent experiments. (f) Comparison of IL-10 production in response to WPG derived from the Bifi-ST or the Bifi-94 strains (*n* = 3). Data are presented as mean ± SD. Statistical analysis was performed using one-way ANOVA followed by Tukey’s post-hoc test. **p < 0.01 and ***p < 0.001 were considered statistically significant.

To identify the effector components of Bifi-94 responsible for stimulating IL-10 production in Breg cells, we treated PEC cells with live, heat-killed, or formalin-fixed Bifi-94, as well as with its culture supernatant, and measured IL-10 levels. Heat-killed and formalin-fixed Bifi-94 induced IL-10 production to levels comparable to those induced by live bacteria. All three Bifi-94 preparations (live, heat-killed, and fixed) stimulated significantly higher IL-10 secretion than the type strain *B. adolescentis* KCTC3216 (Bifi-ST) (Figure 4a). In contrast, culture supernatants from Bifi-94 and Bifi-ST induced comparable IL-10 levels, suggesting that secreted metabolites are not major contributors to the observed effect (Figure 4a).

Consistently, SCFA levels in the culture supernatants did not differ significantly between the two strains (Figures S3a and S3b). To investigate the role of bacterial structural components, we isolated crude bacterial fractions from Bifi-94 according to the experimental scheme shown in Figure S4a. Co-culture of mononuclear cells (Figure 4b) and purified CD11b^+^CD19^+^ Breg cells (Figure 4c) from the PEC with the precipitated bacterial components induced secretion.

Based on these findings, we further purified protein, capsular polysaccharide (CPS), soluble peptidoglycan (SPG), and whole peptidoglycan (WPG) from Bifi-94, components known to modulate immune responses.^35^^,^^36^ When mononuclear cells (Figure 4d) and CD11b^+^CD19^+^ Breg cells (Figure 4e) were co-cultured with these purified components, WPG was the only fraction that significantly induced IL-10 production. We then rigorously assessed the purity of WPG preparation. Teichoic acid (TA) contamination was evaluated by quantifying inorganic phosphate using a colorimetric assay (Figure S4b), and potential lipoteichoic acid (LTA) contamination was examined by visualizing LTA using confocal microscopy (Figure S4c). In addition, possible lipoprotein contaminants were tested functionally by treating WPG preparations with proteinase K (Figure S4d). IL-10 production by mononuclear cells increased in a dose-dependent manner upon stimulation with WPG (Figure S4e). To determine whether this effect was specific to Bifi-94, we also isolated WPG from Bifi-ST and tested its activity. Interestingly, WPG from Bifi-ST induced comparable IL-10 levels to those from Bifi-94 (Figure 4f), indicating that PG itself is the principal effector molecule. These results collectively suggest that WPG is the key bacterial component responsible for promoting IL-10 production in Breg cells.

### *B. adolescentis* Bifi-94 upregulates bacterial PG biosynthesis-related genes and proteins

**Figure 5.**
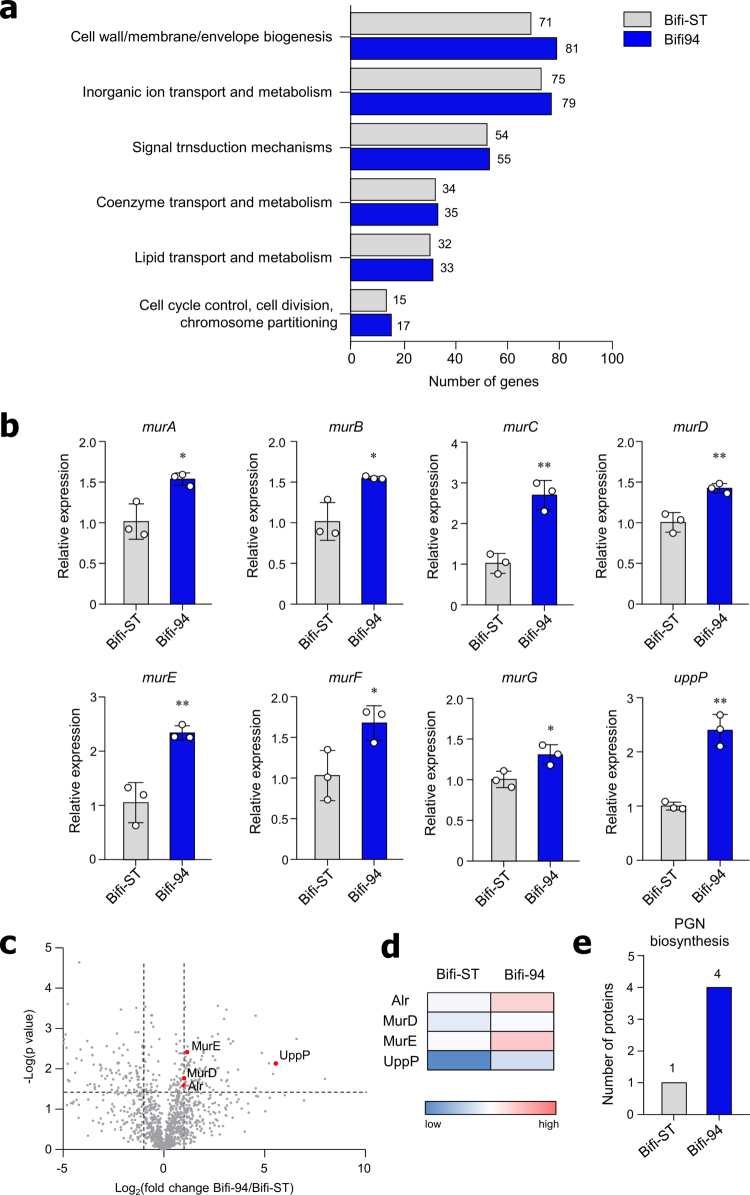
*B. adolescentis* Bifi-94 upregulates genes and proteins related to PG biosynthesis. (a) COGs (Clusters of Orthologous Groups) functional annotation showing a higher number of genes associated with cell wall/membrane/envelope biogenesis in Bifi-94 compared with the Bifi-ST strain. (b) mRNA expression levels of PG biosynthesis-related genes in Bifi-ST and Bifi-94 strains (*n* = 3). (c) Volcano plot and (d) heat map of differentially expressed proteins identified by proteomic analysis. (e) Number of proteins associated with the PG biosynthesis pathway (KEGG pathway ko00550). Data are presented as mean ± SD. Statistical analysis was performed using a two-tailed paired Student’s *t*-test. *p < 0.05 and **p < 0.01 were considered statistically significant.

To investigate the mechanism by which Bifi-94 more effectively enhances IL-10 production in Breg cells compared with the Bifi-ST strain, despite WPGs isolated from both strains inducing similar IL-10 levels, we performed whole-genome sequencing to analyze the genetic characteristics of Bifi-94 (Table S1). Phylogenetic analysis confirmed that Bifi-94 is closely related to Bifi-ST (Figure S5). A complete genome map of Bifi-94 annotated with Clusters of Orthologous Groups (COGs) is presented in Figure S6. Notably, COG functional annotation revealed that the number of genes in the cell wall/membrane/envelope biogenesis cluster was greater in Bifi-94 (*n* = 81) than in Bifi-ST (*n* = 71) (Figure 5a). Based on this observation, we hypothesized that Bifi-94 may exhibit enhanced PG synthesis activity. To test this, we examined the expression of genes involved in PG biosynthesis, including the Mur enzyme family (Figure S7a).^37^ Transcriptomic analysis revealed that Bifi-94 showed higher expression of PG synthesis-related genes, such as *murA*, *murC*, *murD*, *murE*, *murF*, *murG*, and *uppP*, compared with Bifi-ST (Figure 5b).

To evaluate whether these transcriptomic differences translated to the protein level, we performed proteomic analysis. A total of 1,215 and 1,168 proteins were identified from Bifi-ST and Bifi-94, respectively (Figure S7b). Key PG synthesis-related proteins, including Alr, MurE, MurD, and UppP, were upregulated in Bifi-94 relative to Bifi-ST (Figure 5c and 5d). Among the differentially upregulated proteins identified in the two strains, 133 from Bifi-ST and 104 from Bifi-94 were subjected to Kyoto Encyclopedia of Genes and Genomes (KEGG) pathway analysis. Notably, four proteins in Bifi-94 and one in Bifi-ST were associated with the PG biosynthesis pathway (ko00550), suggesting a strain-specific enrichment of this pathway in Bifi-94 (Figure 5e). To further support these findings, we isolated and quantified PG from both strains. The amount of PG tended to be higher in Bifi-94 than in Bifi-ST (Figure S7c). Collectively, these results indicate that Bifi-94 exhibits enhanced PG biosynthesis capacity**,** which may underlie its superior ability to stimulate IL-10 production in Breg cells.

### *B. adolescentis* Bifi-94-derived PG induced IL-10 production by Breg cells in a TLR2-dependent manner

**Figure 6.**
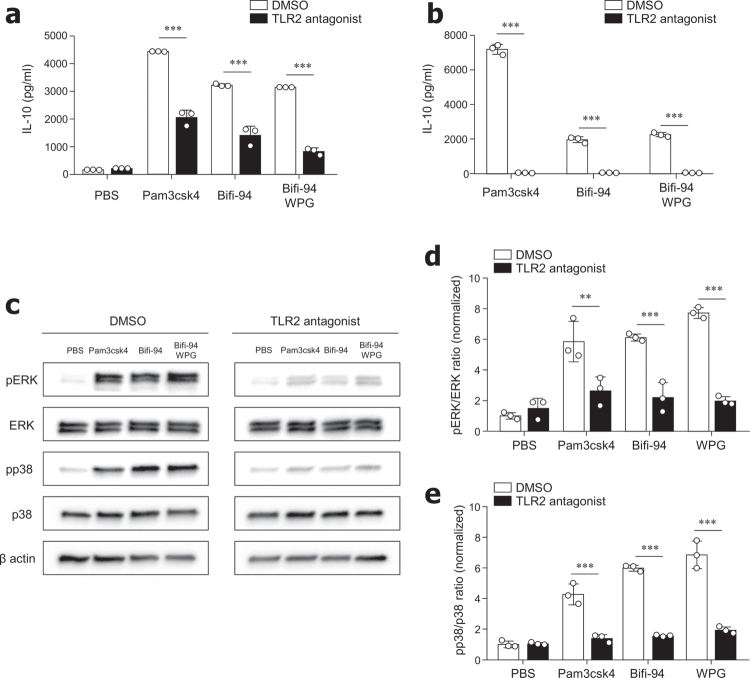
*B. adolescentis* Bifi-94-derived PG induces IL-10 production by Breg cells via a TLR2-dependent manner. (a–b) PEC cells from B6 mice were treated with DMSO or a TLR2 antagonist (200 μM) in the presence of Pam3CSK4 (50 ng/mL), live Bifi-94 (1:10 cell-to-bacteria ratio), SPG (10 μg/mL), or WPG (10 μg/mL) for 72 hours (*n* = 3). IL-10 levels in the culture supernatants were quantified by ELISA for (a) total PEC cells and (b) sorted CD11b^+^CD19^+^ Breg cells. (c–e) PEC cells were pretreated with DMSO or a TLR2 antagonist (200 μM) for 2 hours prior to stimulation with Pam3CSK4, Bifi-94, or Bifi-94-derived WPG for 30 minutes. Cell lysates were analyzed by Western blotting for phosphorylated and total ERK (pERK, ERK) and p38 MAPK (pp38, p38). (c) Representative Western blot images showing pERK, ERK, pp38, and p38 expression (*n* = 3). (d–e) Quantification of pERK and pp38 phosphorylation, normalized to total ERK and p38, respectively (*n* = 3). Data are presented as mean ± SD and were combined from ≥3 independent experiments. Statistical analysis was performed using one-way ANOVA followed by Tukey’s post-hoc test. *p < 0.05, **p < 0.01 and ***p < 0.001 were considered statistically significant.

Given that TLR2 plays a key role in recognizing PG and initiating downstream immune responses,^38^^,^^39^ we examined whether Bifi-94-induced IL-10 production in Breg cells depends on TLR2 signaling. PEC-derived mononuclear cells and CD11b⁺CD19⁺ Breg cells were treated with a TLR2 antagonist prior to stimulation with Bifi-94. Of note, TLR2 inhibition markedly reduced IL-10 production in mononuclear cells and almost completely abolished IL-10 secretion in purified Breg cells, indicating that TLR2 signaling plays a central role in Bifi-94–induced IL-10 responses. Consistently, IL-10 production induced by Pam3CSK4, a TLR2 agonist used as a positive control, was also efficiently suppressed by the TLR2 antagonist under the same conditions, confirming effective TLR2 blockade in our system (Figure 6a and 6b). Previous studies have demonstrated that activation of ERK and p38 MAPK pathways downstream of TLR2 promotes IL-10 production in macrophages and DCs.^40-42^

To determine whether these pathways are also involved in Bifi-94-mediated Breg cell activation, we examined ERK and p38 phosphorylation in PEC-derived mononuclear cells stimulated with either whole Bifi-94 and purified WPG (Figure 6c, 6d and 6e). Both Bifi-94 and Bifi-94-derived WPG robustly activated ERK and p38 phosphorylation (Figure 6c, 6d and 6e). Importantly, pre-treatment with the TLR2 antagonist completely suppressed this activation, confirming that the ERK and p38 pathways are activated downstream of TLR2 in response to Bifi-94 and its WPG component. Western blot images are available in Figure S8. Together, these findings demonstrate that WPG derived from Bifi-94 stimulates IL-10 production in CD11b⁺CD19⁺ Breg cells through TLR2-dependent activation of the ERK and p38 MPK signaling pathways.

### *B. adolescentis* Bifi-94 induces IL-10 production by B cells in the human gut

**Figure 7.**
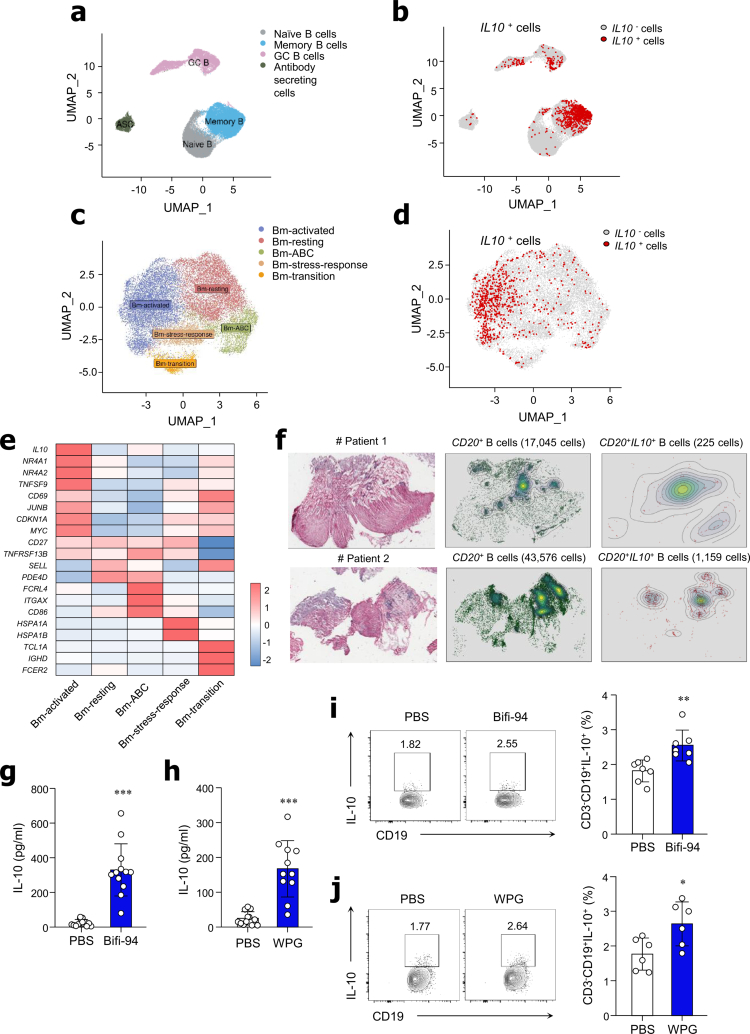
*B. adolescentis* Bifi-94 induces IL-10 production by B cells in the human gut. (a) UMAP (Uniform Manifold Approximation and Projection) plot showing clustering of *CD19*^*+*^ B cells. (b) UMAP plot showing segregation of *IL10⁺* and *IL10^–^* B cells based on *IL10* expression. (c) UMAP plot of memory B cell subclusters. (d) UMAP representation of *IL10⁺* and *IL10^–^* memory B cells. (e) Heat map showing relative gene expression profiles of memory B cell subclusters. (f) H&E-stained image of small intestinal tissue analyzed using the 10x Xenium platform, with accompanying spatial density plots of *CD20*^*+*^ and *CD20*^*+*^*IL-10*^*+*^ B cells (*n* = 2). (g–j) Mononuclear cells from human colon tissue were co-cultured with Bifi-94 (1:10 ratio) or WPG (10 μg/mL) for 72 hours. (g–h) IL-10 levels in culture supernatants following co-culture with Bifi-94 or WPG were quantified by ELISA (*n* = 11−12). (i–j) IL-10 expression in CD19^+^ B cells was assessed by flow cytometry, and the frequency of IL-10^+^ B cells were determined after co-culture with (i) Bifi-94 or (j) WPG (*n* = 6−7). Data are presented as mean ± SD. Statistical analysis was performed using a two-tailed paired Student’s *t*-test. *p < 0.05, **p < 0.01 and ***p < 0.001 were considered statistically significant.

To determine whether IL-10-producing B cells are present in the human intestine, we analyzed publicly available single-cell RNA-sequencing (scRNA-seq) data from small intestinal tissues of both Crohn’s disease patients and healthy individuals. B cell clusters were extracted for further analysis and categorized into four distinct subsets: naïve B cells, memory B (Bm) cells, germinal center B cells, and antibody-secreting cells (Figure 7a). Among these, IL-10 expression was predominantly enriched in the Bm cells cluster (Figure 7b). We then performed sub-clustering analysis on the Bm cell population, identifying five transcriptionally distinct subsets: Bm-activated, Bm-resting, Bm-atypical B cells (Bm-ABC), Bm-stress-response, and Bm-transition subsets (Figure 7c). IL-10 expression and the proportion of IL-10^*+*^ cells were highest in the Bm-activated subset, suggesting this population may represent a regulatory B cell subset in the human intestine (Figure 7d and 7e).

To further validate these findings in tissue, we performed spatial transcriptomic analysis on small intestinal sections from two patients with Crohn’s disease (Figure 7f). *CD20^+^* B cells were found to be distributed throughout the intestinal tissue, and a subset of these cells expressed IL-10, indicating the presence of IL-10-producing B cells within the gut microenvironment (Figure 7f). These results suggest that are immunoregulatory B cells exist in human gut tissues and may play a role in mucosal immune regulation. To assess whether Bifi-94 stimulates IL-10 production by human gut B cells, we isolated mononuclear cells from normal colonic tissues obtained from surgical patients with various clinical backgrounds (Table S2). Cells were cultured with Bifi-94 and Bifi-94-derived WPG. Supernatants from cultures treated with Bifi-94 (Figure 7g) or its WPG (Figure 7h) exhibited increased levels of IL-10 compared with controls. Flow cytometric analysis also showed an increased frequency of CD19^+^IL-10^+^ cells after treatment with Bifi-94 (Figure 7i) or WPG (Figure 7j). These results indicate that Bifi-94 and its WPG component can enhance IL-10 production by B cells in human colon tissues.

## Discussion

In this study, we identified that *B. adolescentis* Bifi-94, a novel strain isolated from human feces, significantly promotes IL-10 production by Breg cells, but not by Treg cells. Among the cell wall components derived from Bifi-94, PG was the key effector molecule driving IL-10 induction in Breg cells. This effect was mediated through TLR2-dependent activation of the ERK and p38 MAPK signaling pathways. Transcriptomic and proteomic analyzes further revealed that genes and proteins involved in PG biosynthesis were upregulated in Bifi-94 compared with a type strain. Bifi-94 also stimulated IL-10 production in human B cells, and the presence of *IL-10⁺* B cells in the gut lamina propria was confirmed through scRNA-seq and spatial transcriptomics. Together, these findings indicate that Bifi-94-derived PG activates TLR2 signaling in Breg cells to promote IL-10 production and mucosal immune regulation (Figure S9).

Previous studies have shown that the gut microbiota promotes IL-10 production in a range of immune cell types, thereby maintaining immune tolerance and preventing excessive inflammation.^43^^,^^44^ Specific gut microbes such as *Bifidobacterium breve* and *Clostridium* species enhance immune tolerance by inducing IL-10-producing Foxp3^+^ Treg cells, offering protection against intestinal inflammation.^45^^,^^46^ Other studies have demonstrated that adherent strains of *E. coli* can increase IL-10 secretion by macrophages, reducing intestinal inflammation and lowering the risk of colitis-associated colorectal cancer.^47^ Similarly, outer membrane vesicles derived from *Bacteroides thetaiotaomicron* have been shown to promote IL-10 expression in both colonic and peripheral DCs.^48^ Furthermore, commensal bacteria residing in the gut have been implicated in the induction of IL-10-producing B cells within the intestinal mucosa.^19^ In line with these reports, our data show that *B. adolescentis* Bifi-94 drives IL-10 production specifically in Breg cells, supporting the concept that commensal bacteria regulate mucosal immune responses by promoting IL-10 across multiple immune lineages.

Consistent with previous reports that IL-10–producing Breg cells exert potent anti-inflammatory effects in the intestine, our findings demonstrate that *B. adolescentis* Bifi-94 and its PG enhance IL-10 production and ameliorate DSS-induced colitis.^19^^,^^49^ This induction of IL-10–expressing Breg cells likely contributes to the observed mucosal protection and suppression of pro-inflammatory cytokines. Although our results strongly suggest that IL-10–producing B cells mediate the protective effects of Bifi-94, the causal role of IL-10 has not been directly demonstrated. Future studies employing IL-10-neutralizing antibodies or IL-10–deficient mice will be essential to confirm that IL-10 is the key mediator of Bifi-94-induced immunoregulation. Nevertheless, the consistent upregulation of IL-10–related genes and the expansion of IL-10⁺ B cells across multiple tissues provide compelling correlative evidence that IL-10 signaling underlies the observed protection.

Growing evidence suggests that Breg cells are critical for maintaining immune tolerance and regulating inflammatory responses. For example, CD19^–/–^ mice, which lack key Breg subsets including B10 B cells, develop more severe DSS-induced colitis.^49^ Intestinal B-1 B cells also have been shown to suppress colitis through the production of IgA.^23^ Building on these findings, our data demonstrate that Bifi-94 mitigates intestinal inflammation by inducing IL-10-producing Breg cells, underscoring their central role in mucosal immune homeostasis. Notably, Breg cells are also implicated in the regulation of systemic autoimmune diseases, such as in EAE.^50^^,^^51^ A recent study revealed that TIM1^+^ B cells express co-inhibitory molecules such as TIGIT, and that the TIM1-AhR-TIGIT axis is crucial for maintaining immune tolerance and preventing autoimmunity.^52^ Collectively, these insights raise the possibility that Bifi-94 may modulate both intestinal and systemic inflammation through activation of distinct Breg subsets, including B-1 B cells.

Breg cells have been identified in multiple tissues.^49^^,^^53^^,^^54^ Our phenotypic analysis of IL-10-producing B cell subsets, including B-1a cells (CD19⁺CD5⁺), B10 cells (CD19⁺CD1d⁺CD5⁺), TIM-1⁺ B cells (CD19⁺TIM-1⁺), and CD9⁺ B cells (CD19⁺CD9⁺), revealed that these populations were most enriched in the PEC (Figure S10). Although B220^low/mid^CD19^+^ Breg cells were abundant in Peyer’s patches, they did not produce IL-10 in response to Bifi-94 stimulation, suggesting that the immunoregulatory function of Breg cells may be influenced by their anatomical location (Figure S2e and S2f). These data support the idea that Breg cells exhibit functional heterogeneity across tissues and that their cytokine output—particularly IL-10 production—is shaped by the local microenvironment.

Microbiota-derived metabolites and cell wall components are increasingly recognized as key anti-inflammatory agents that act through TLR2-mediated induction of IL-10. For instance, cell wall components such as TA and LTA from *Lactobacillus plantarum* stimulate IL-10 production via TLR2-ERK signaling in macrophages, highlighting the strain-specific nature of microbial immunomodulation.^55^ Likewise, LPS from *Akkermansia muciniphila* and PSA from *Bacteroides fragilis* induce IL-10 expression through TLR2-dependent pathways.^56^^,^^57^ Our results extend this paradigm by showing that Bifi-94 promotes IL-10 production in B cells through TLR2-dependent MAPK activation. Together, these results support a broader concept in which microbial metabolites and cell wall components orchestrate anti-inflammatory responses across distinct immune cell types by engaging TLR2 signaling cascades. While our findings demonstrate that pharmacological inhibition of TLR2 completely abrogates Bifi-94– and PG-induced IL-10 production and MAPK activation, future validation using TLR2-deficient mice or cells will be crucial to definitively establish the receptor’s role.

PG is increasingly recognized as a bioactive immunomodulatory molecule with diverse effects ranging from inflammation to immune suppression and anti-tumor activity.^38^^,^^58^ The immunoregulatory capacity of Bifi-94 likely reflects strain-specific PG structures, including modifications of the glycan backbone or peptide stem.^59^^,^^60^ In *B. adolescentis*, anhydro-PG structures containing anhydro-*N*-acetylmuramic acid termini constitute nearly 40% of total PG and have been reported to suppress pro-inflammatory cytokines such as TNF-*α*, IL-6, and IL-1β.^58^ It is plausible that similar structural motifs contribute to the anti-inflammatory effects of Bifi-94. Although compositional profiling has not yet been performed, our proteomic and transcriptomic data revealed an upregulation of PG biosynthetic enzymes in Bifi-94 relative to Bifi-ST. These findings, together with the selective activation of the TLR2–ERK/p38 pathway by purified PG, indicate that subtle structural variations or enrichment of specific PG motifs may contribute to the enhanced immunoregulatory properties of Bifi-94.

Our results show that oral administration of Bifi-94 increases the number of Breg cells in the PEC and enhances their IL-10 production. A previous study demonstrated that oral LPS activates B-1 B cells not only in the intestinal lamina propria but also in the PEC,^61^ suggesting that B-1 B cells in these compartments may constitute a shared, responsive pool capable of sensing enteric stimuli and migrating between mucosal and peritoneal tissues. Similarly, intraperitoneal injection of CD19^+^ B cells or intravenous transfer of PEC, which are composed predominantly of B cells and macrophages, has been shown to ameliorate DSS-induced colitis.^54^^,^^62^ These findings imply that peritoneal mononuclear cells can migrate to inflamed intestinal tissue and exert local immunoregulatory effects. Thus, the anti-inflammatory activity of Bifi-94 may be mediated by migratory Breg cells that bridge the PEC and intestinal mucosa.

Recent studies have identified IL-10-producing B cells in humans that play key roles in immune regulation. For example, CD24^hi^CD38^hi^ B cells in human PBMCs produce IL-10 and suppress Th1 differentiation,^22^ while CD24^hi^CD38^hi^CD27^+^ transitional B cells secrete IL-10 and inhibit pro-inflammatory cytokine production.^63^ Among these subsets, B10 cells—characterized by robust IL-10 production—are typically CD24^hi^CD27^+^ and phenotypically resemble Bm cells.^64^ In our scRNA-seq dataset, IL-10 expression was most enriched within the Bm cell cluster, particularly in the Bm-activated subcluster that exhibited the highest CD27 expression. This transcriptional signature closely mirrors that of human B10 cells, suggesting that a B10-like regulatory subset resides within the IL-10–enriched Bm compartment. A limitation of our study is the small sample size used for the spatial transcriptomics analysis (two Crohn’s disease patients), which may limit generalizability. Nevertheless, the consistent spatial localization and transcriptional features across samples support the robustness of these observations.

In conclusion, we demonstrate that *B. adolescentis* Bifi-94 exerts potent immunomodulatory effects by promoting IL-10 production, particularly in Breg cells. Importantly, this IL-10-inducing activity was also observed in human B cells, underscoring the strain’s translational relevance. These findings highlight *B. adolescentis* Bifi-94 as a promising therapeutic candidate for the treatment of inflammatory diseases.

## Materials & methods

### Ethics statement

All animal experiments were approved by the Institutional Animal Care and Use Committee of the Asan Institute for Life Sciences (Approval No. 2022-12-230 and 2024-20-129). Human fecal and intestinal tissue samples were obtained from the Human Dock Center of Asan Medical Center with approval from the Institutional Review Board (Approval No. A20201614 and 2019-1294). All procedures were conducted in accordance with the relevant institutional guidelines and regulations.

### Isolation of *Lactobacillus* and *Bifidobacterium* strains from human stool

A total of 88 human fecal samples were collected from the Human Dock Center at Asan Medical Center, using fresh residual material obtained on the same day as routine examinations for fecal occult blood and parasites. Fecal samples were suspended in PBS and plated onto MRS agar (BD Biosciences, Franklin Lakes, NJ) supplemented with vancomycin to isolate *Lactobacillus* strains, and onto TOS-MUP agar (Kisan-Bio, Seoul, Korea) to isolate *Bifidobacterium* strains. Plates were incubated at 37°C under anaerobic conditions using the GasPak 100 system (BD Biosciences). Approximately 300 colonies from MRS agar and 100 colonies from TOS-MUP agar were randomly selected within the countable range (30–300 CFU/plate) to ensure representative isolation. Colony PCR targeting the *Lactobacillus* and *Bifidobacterium* genera was performed using the following primer sets: 5′-AGCAGTAGGGAATCTTCCA-3′ and 5′-ATTYCACCGCTACACATG-3′ for *Lactobacillus*, and 5′-TGGCAGTCCAACAAGCRC-3′ and 5′-TAGGAGCTCCAGATGCCGTG-3′ for *Bifidobacterium.*^6^^,^^65^ PCR was conducted on a Veriti thermal cycler (Applied Biosystems, Foster City, CA) (*Lactobacillus*: 95 °C for 5 min; 35 cycles of 95 °C for 30 s, 54 °C for 30 s, 72 °C for 60 s; and final extension at 72 °C for 7 min; *Bifidobacterium*: 95 °C for 3 min; 35 cycles of 95 °C for 30 s, 58 °C for 30 s, 72 °C for 60 s; and final extension at 72 °C for 5 min), and products were analyzed on 1.5% agarose gels using a Gel Doc XR + imaging system (Bio-Rad).

### Mice

Six-week-old female C57BL/6 (B6) mice were purchased from OrientBio (Seongnam, Korea). Mice were housed under specific pathogen-free conditions in the animal facility of Asan Medical Center and provided sterile food and water *ad libitum*. *Bifidobacterium adolescentis* strains [Bifi-94 (KCTC 16376BP) and Bifi-ST strains (KCTC3216)] were suspended in 300 μL of anaerobic PBS at a dose of 3 × 10⁹ CFU and administered orally once daily for 2 or 4 weeks, depending on the experimental protocol.

### DSS-induced acute colitis model

Each *Bifidobacterium* strain was cultured in MRS broth under anaerobic conditions for 24 h, harvested by centrifugation, washed twice with sterile anaerobic PBS, and resuspended in 300 μL of anaerobic PBS at a final dose of 3 × 10^9^ CFU. Before colitis induction, the bacterial suspension was orally administered to mice once daily for 2 weeks. Acute colitis was induced by providing 3.5% DSS (w/v) (molecular weight 36,000–50,000, MP Biomedicals) in the drinking water for 6 consecutive days, followed by regular tap water for 1 day. Body weight was monitored daily to assess disease severity throughout the experimental period. The total DAI scroe was calculated as the average of the scores for body weight loss, stool consistency, and stool blood.^66^

### *In vitro* screening

To screen for bacterial strains capable of promoting IL-10 production, total mononuclear cells were isolated from the spleens of B6 mice. To assess IL-12p70 production, bone marrow-derived DCs were also prepared from B6 mice. These immune cells were co-cultured with *Lactobacillus* and *Bifidobacterium* strains at a cell-to-bacteria ratio of 1:10 in complete RPMI medium. The medium comprised RPMI 1640 (Gibco) supplemented with 10% heat-inactivated fetal bovine serum (FBS; Gibco), penicillin/streptomycin (Gibco), 2 mM L-glutamine (Gibco), 1 mM sodium pyruvate (Gibco), 0.1 mM non-essential amino acids (Gibco), 10 mM HEPES buffer (Gibco), and 50 μM 2-mercaptoethanol (Sigma-Aldrich). To limit bacterial overgrowth during co-culture, gentamicin (150 μg/mL, Gibco) was added to the complete RPMI medium. After 72 hours of incubation, culture supernatants were collected, and cytokine concentrations were determined by ELISA. IL-10 levels were measured using a mouse IL-10 ELISA kit (Invitrogen, Carlsbad, CA), according to the manufacturer’s instructions, whereas IL-12p70 levels were determined by a manually established sandwich ELISA. Sample were added to 96-well microplates pre-coated with an anti-IL-12p70 capture antibody (clone 48110; R&D Systems, Minneapolis, MN) and incubated at room temperature for 2 h. After washing, biotinylated anti-IL-12 detection antibody (clone P43432; R&D Systems) and streptavidin-HRP were sequentially applied. Cytokine concentrations were normalized to viable cell counts.

### Histology

Colon tissues were opened longitudinally, rolled from distal to proximal ends, and fixed in 4% paraformaldehyde (PFA) for 24 hours at room temperature. Fixed tissues were paraffin-embedded, sectioned, and stained with hematoxylin and eosin (H&E). Histopathological scoring was performed based on three parameters: inflammation (0, none; 1, slight; 2, moderate; 3, severe), extent of injury (0, no injury; 1, mucosal involvement only; 2, involvement of mucosa and submucosa; 3, full-thickness epithelial loss), and crypt damage (0, none; 1, basal one-third damaged; 2, basal two-thirds damaged; 3, only surface epithelium intact; 4, entire crypt lost). Each score was multiplied by a factor corresponding to the percentage of tissue involved (1, 1–25%; 2, 26–50%; 3, 51–75%; 4, 76–100%). The total histology score was calculated as the sum of these weighted parameter scores.

### Treg differentiation assay

Naïve CD4⁺ T cells were isolated from the spleens of B6 mice using a naïve CD4⁺ T Cell Isolation Kit (Miltenyi Biotec), according to the manufacturer’s protocol. Purified cells were cultured at a density of 1–2 × 10^5^ cells/mL in complete RPMI medium. For T cell activation, 96-well flat-bottom plates were pre-coated with anti-CD3ε monoclonal antibody (1 μg/mL, clone 145-2C11, BioLegend, San Diego, CA) for 2 hours at 37°C. Soluble anti-CD28 monoclonal antibody (1 μg/mL, clone 37.51, BioLegend) was added at the start of the culture. Cells were maintained under Treg-polarizing conditions in the presence of recombinant mouse IL-2 (10 ng/mL) and human TGF-*β* (0.2 ng/mL) (both from R&D Systems) for 3 days at 37°C in a humidified 5% CO₂ incubator.

### Primary cell isolation from mice and humans

To isolate mononuclear cells, colons from mice and human donors were opened longitudinally, washed with cold PBS, and cut into approximately 1-cm pieces. Tissues were incubated in RPMI containing 1 mM EDTA at 37°C for 20 minutes. After incubation, samples were vigorously shaken in pre-warmed PBS, then minced and digested in RPMI supplemented with collagenase D (500 μg/mL; Sigma-Aldrich, St. Louis, MO) and DNase I (100 μg/mL; Roche, Basel, Switzerland) at 37°C for 30 minutes. Mononuclear cells were isolated by density gradient centrifugation with 40% and 75% Percoll (Cytiva, Marlborough, MA). PEC cells were collected by injecting 5 mL of PBS containing 3% FBS into the PEC, followed by gentle massage and aspiration of the fluid. For Peyer’s patches, tissues were excised from the small intestine, washed in PBS, digested in RPMI containing collagenase D and DNase I at 37°C for 30 minutes, then minced and passed through 100 μm and 40 μm cell strainers. Spleens were minced and passed through the suspension through a 100 μm strainer, and red blood cells were lysed using ACK lysis buffer (Gibco, Grand Island, NY) at 4°C. Final cell suspensions were filtered through a 40 μm cell strainer before downstream application.

### Cell culture

Isolated mononuclear cells were cultured in complete RPMI medium. Cells were plated in 96-well flat-bottom plates and co-cultured with live, heat-killed, fixed bacteria, or bacterial culture supernatants. Heat-killed bacteria were prepared by incubation at 95°C for 30 minutes. Fixed bacteria were generated by treating bacterial suspensions with 4% paraformaldehyde (PFA) for 20 minutes, followed by two washes with PBS. Bacterial culture supernatants were collected by centrifugation at 12,000 rpm for 10 minutes and sterilized by filtration through a 0.22 μm syringe filter (Pall Corporation, Port Washington, NY). Live, heat-killed, and fixed bacteria were added to mononuclear cells at a cell-to-bacteria ratio of 1:10. Bacterial culture supernatants were added at a final concentration of 2% and incubated for 72 hours. Additionally, bacterial cell components, including protein, CPS, and PG, were added at a final concentration of 10 μg/mL and incubated under the same conditions. For TLR2 inhibition experiments, PEC cells or sorted Bregs from B6 mice were treated with DMSO or a TLR2 antagonist TL2-C29 (200 μM; Invivogen, San Diego, CA) in the presence of Pam3CSK4 (50 ng/mL), live Bifi-94 (1:10 cell-to-bacteria ratio), SPG (10 μg/mL), or WPG (10 μg/mL) for 72 hours.

### Flow cytometry

Isolated mouse mononuclear cells were stimulated for 4 hours with PMA (50 ng/mL; Sigma-Aldrich), ionomycin (1 μg/mL; Sigma-Aldrich), LPS (10 μg/mL; Sigma-Aldrich), and a protein transport inhibitor cocktail containing Brefeldin A and Monensin (Invitrogen). After stimulation, cells were washed with PBS containing 1% FBS and blocked with anti-CD16/32 antibody (BD Biosciences). Surface marker staining was performed using fluorophore-conjugated antibodies in conjunction with a Live/Dead Cell Stain kit (Thermo Fisher Scientific, Waltham, MA). Intracellular staining was conducted using a cytofix/cytoperm fixation/permeabilization kit (BD Biosciences) for cytokine detection, whereas intracellular Foxp3 staining was carried out using the Foxp3/transcription factor staining buffer set (Invitrogen). The following antibodies were used for mouse samples: anti-CD45 (30-F11), anti-CD4 (RM4.5), anti-B220 (RA3-6B2), anti-CD11b (M1/70), anti-Foxp3 (FJK-16s), anti-CD19 (1D3), and anti-TIM-1 (RMT1-4) from BD Biosciences; and anti-CD3 (17A2), anti-IL-10 (JES5-16E3), anti-CD9 (MZ3), anti-CD5 (53-7.3), and anti-CD1d (1B1) from Biolegend. Human mononuclear cells from colon tissue were stimulated with CpG oligodeoxynucleotides (10 μg/mL; Invivogen); sequence: 5′-tcgtcgttttgtcgttttgtcgtt-3′), live bacteria (1:10 cell-to-bacteria ratio), or WPG (10 μg/mL) for 72 hours. Cells were then restimulated with PMA, ionomycin, and GolgiPlug (BD Biosciences) for 4 hours and stained as described above. The following antibodies used were for human samples: anti-CD45 (HI30), anti-CD3 (UCHT1), and anti-CD19 (HIB19) from Invitrogen; and anti-IL-10 (JES3-19F1) from BD Biosciences.

### Extraction and purification of bacterial cell components

*B. adolescentis* was cultured anaerobically in MRS broth supplemented with 0.05% (w/v) L-cysteine HCl at 37°C for 24 hours. For CPS isolation, bacterial cells were harvested, washed, resuspended in distilled water, and sonicated (30 minutes, 40% amplitude, 10 s on/off cycles). The lysate was centrifuged, and the supernatant was treated with 0.5% (w/v) trichloroacetic acid (TCA) at 4°C overnight to remove proteins. After centrifugation, the supernatant was precipitated with cold ethanol (ethanol:sample ratio of 3:1) at −20°C overnight. The resulting pellet was sequentially digested with RNase (0.4 mg/mL, Worthington Biochemical Corporation, Lakewood, NJ) and DNase (0.1 mg/mL, Roche), and pronase (0.3 mg/mL, Merck Millipore, Burlington, MA), followed by protein removal with 2% TCA. The final polysaccharide product was precipitated with ethanol, resuspended in distilled water, dialyzed using a 10 kDa molecular weight cut-off (MWCO) membrane for 3 days, and lyophilized. CPS concentration was quantified using the phenol-sulfuric acid method.^67^ In parallel, PG was purified from bacterial cell walls following previously described procedures^68^ with minor modifications. The overall workflow was based on standard purification methods involving SDS extraction, enzymatic digestion, and acid treatment to remove non-PG components.^69^ Bacterial pellets were sonicated for 30 minutes (40% amplitude, 10 s on/off cycles) in 1 M NaCl to remove loosely bound proteins, treated with 0.5% SDS at 60°C for 30 minutes to solubilize membrane-associated materials and lipoproteins, and then washed extensively. The resulting pellet was digested with RNase (50 μg/mL), DNase (10 μg/mL), and trypsin (200 μg/mL, Sigma-Aldrich) in 1 M Tris-HCl (pH 6.8) to degrade residual nucleic acids and protein contaminants. After centrifugation, the pellet was incubated in 5% TCA to extract TA, washed with 100% acetone, and lyophilized to obtain purified whole PG. Soluble PG was prepared by enzymatic digestion using mutanolysin (500 U, Sigma-Aldrich) at 37°C overnight. To confirm the absence of TA contamination, inorganic phosphate levels were measured using a pyrophosphate assay kit (invitrogen), with purified LTA (10 μg/mL, Sigma-Aldrich, L3265) included as a positive control. To confirm the absence of lipoprotein contamination, PG preparations (WPG and SPG) were treated with proteinase K (100 μg/mL, Sigma-Aldrich) in 100 mM Tris-HCl (pH 8.0) containing 2 mM CaCl_2_ at 37°C for 2 hours, followed by enzyme inactivation by boiling for 10 min. Cell wall-associated proteins were recovered from the TCA supernatant during CPS extraction, neutralized with NaOH, and quantified using the BCA protein assay (Thermo Fisher Scientific).

### Genomic DNA extraction and whole-genome sequencing

Genomic DNA (gDNA) from the Bifi-94 and Bifi-ST strains was extracted using the MagAttract HMW DNA Kit (Qiagen, Hilden, Germany), following the manufacturer’s instructions. DNA integrity was assessed by agarose gel electrophoresis, and concentrations were determined using a Qubit 2.0 fluorometer (Invitrogen). A total of 5 μg of gDNA was sheared using the Megaruptor3 system (Diagenode), and sequencing libraries were prepared using the SMRTbell Express Template Preparation Kit (PacBio), optimized for 20 kb templates. Library quality and concentration were assessed using the Qubit fluorometer and the Bioanalyzer high-sensitivity DNA chip (Agilent Technologies). Sequencing was performed using PacBio Sequel Sequencing Kit v3.0 and SMRT Cell 1 M v2. De novo genome assemblies were generated using PacBio SMRT Analysis software suite and analyzed by CJ Bioscience, Inc. Assembled contigs were circularized using Circlator 1.4.0. Gene prediction and functional annotation were performed using the EzbioCloud genome database. Protein-coding sequences (CDSs) were predicted using Prodigal 2.6.2. tRNA genes were identified using tRNAscan-SE, and rRNAs and other non-coding RNAs were annotated via comparison with the Rfam database. Comparative whole-genome analysis between Bifi-94 and Bifi-ST was conducted using nucleotide identity by BLAST (ANIb), calculated with the ANI calculator provided by the Kostas lab (http://enve-omics.ce.gatech.edu/ani).

### RNA extraction and quantitative real-time PCR

Total RNA was extracted from bacterial cells using the TRIzol reagent (Thermo Fisher Scientific) according to the manufacturer’s instructions. RNA purity and concentration were determined by measuring the A260/A280 ratio using a NanoDrop 2000 spectrophotometer (Thermo Fisher Scientific). For cDNA synthesis, 500 ng of total RNA was reverse-transcribed using the ReverTra Ace qPCR RT Master Mix with gDNA Remover (Toyobo, Japan) following the manufacturer’s manual. Quantitative real-time PCR was performed on a 7500 Real-Time PCR System (Applied Biosystems) using SYBR Green Master Mix (Thermo Fisher Scientific). Thermal cycling conditions were 95 °C for 2 min, followed by 40 cycles of 95 °C for 15 s, 60 °C for 15 s and 72 °C for 60 s, with a final melt curve analysis (65–95 °C, +1 °C increments). Primers for target genes were designed using Primer-BLAST (https://www.ncbi.nlm.nih.gov/tools/primer-blast/). The primer sequences used were as follows: *murA*, 5′-AAGGAACACAAGGACGGCAT-3′ and 5′-CACGCTCACCAGATCCATGA-3′; *murB*, 5′- CGGCTTCCATAAGGGGTTCA-3′ and 5′- GGGCGATATCTTCGGCTGAT-3′; *murC*, 5′- GCAGTCCAAGATCCGTGTGA-3′ and 5′- GGGAAGATGCCGGTAACGAT-3′; *murD*, 5′- AGAAGGCGCTGGTCTACAAC-3′ and 5′- TCTTTCACACCGATCTGCCC-3′; *murE*, 5′- CCTCGTATTGGGTGCCGATT-3′ and 5′- TCGATGACGATGGAGAAGCG-3′; *murF*, 5′- CTGCTCAAATCGCTGCTGTC -3′ and 5′- GAACCAGGGAGGTGAGGTTG-3′; *murG*, 5′- AAGGGCAAAGACGACGAAGT-3′ and 5′- CGTTCCAGATAGGGAGCCAC-3′; *uppP*, 5′- GTTCATCATCATCGGCACGC-3′ and 5′- AGATCAGCGCATCCTTCCAG-3′; *Tuf* (elongation factor Tu), 5′-CGTGACCTCCTCGACGAAAA-3′ and 5′-ATCAGGAACGGCTTGTCCAG-3. Each reaction was performed in triplicate. The specificity of amplification was confirmed by a single-peak melt curve and a band on agarose gel electrophoresis. Gene expression levels of PG biosynthesis-related genes were calculated using the 2^–ΔΔ Ct^ method.^70^ Expression levels were normalized to the internal reference gene *Tuf*, which exhibited stable Ct values across samples.^71^ The Bifi-ST strain was used as the calibrator for relative comparison.

### Preparation of bacterial cell membrane sample for proteome analysis

The bacterial cell membrane fractions for each bacterium was lyophilized and was suspended with 5% sodium dodecyl sulfate (SDS) buffer with 50 mM triethylammonium bicarbonate (TEAB) (pH 8.5) for denaturation. Dithiothreitol (DTT) of 20 mM was treated on the lysates to reduce disulfide bonds on proteins and the samples were incubated at 95°C for 30 minutes. Iodoacetamide (IAA) of 40 mM was added and incubation in the dark at room temperature for 30 minutes was followed for alkylation of cysteine residues on protein. For acidification for digestion conditioning, 10-fold dilution of 12% phosphoric acid was added and suspension trap digestion (S-Trap) binding buffer (90% methanol, 100 mM TEAB (pH 7.55)) was added to 500 µL as described by manufacture. After washing the S-Trap spin column (ProtiFi, Long Island, New York) with 150 µL of S-trap binding buffer and centrifuging at 4,000 g for 30 seconds, the washing step was repeated two times. A trypsin/Lys-C mix with a protein to Trypsin/Lys-C mixture ratio of 50:1 (Promega, Madison, WI) dissolved in 50 mM TEAB (pH 8.5) was added to the spin column for digestion reaction. The column was incubated at 37°C for 12 hours without shaking.^72^ Peptides was eluted by adding 40 µL of 50 mM TEAB, centrifuging at 1,000 g for 1 minute, and then by adding 40 µL of 0.2% formic acid, followed by centrifugation at 1,000 g for 1 minute. For the final elution step, 40 µL of 0.2% formic acid and 50% acetonitrile were added, and the sample was centrifuged at 4,000 g for 1 minute. The peptides were dried and stored at –80°C until LC-MS analysis.

### LC-MS analysis and database search

Peptide mixture was reconstituted in 0.1% formic acid with 0.005% *n*-dodecyl-beta-D-maltoside (DDM). Peptide separation was performed using Ultimate3000 RSLC system coupled with a Q Exactive HFx mass spectrometer (Thermo Fisher Scientific). The liquid chromatography gradient and data-dependent acquisition-MS options followed previously published methods.^73^ The acquired MS spectra were searched using Sequest HT on Proteome Discoverer (version 2.4, Thermo Fisher Scientific) against the proteome sequence FASTA files (*Bifidobacterium adolescentis (strain KCTC3216/ATCC 15703/DSM 20083/NCTC 11814/E194a)* from Uniprot (https://www.uniprot.org/proteomes), respectively. Fixed modification at cysteine for carbamidomethylation was applied. Resulting identification and label free quantities of proteins in each sample set was extracted and was used for further analysis.

### Western blot

Total mononuclear cells from the PEC of B6 mice were isolated and seeded at 5 × 10⁶ cells per well in 6-well plates. After 24 hours, cells were pretreated with either DMSO or a TLR2 antagonist for 2 hours, followed by stimulation with Bifi-94 (cell-to-bacteria ratio 1:10), WPG (10 µg/mL), or Pam3CSK4 (50 ng/mL) for 30 minutes. Cells were then lysed in RIPA buffer (Thermo Fisher Scientific) supplemented with 1% Halt Protease and Phosphatase Inhibitor Cocktail (Thermo Fisher Scientific). Proteins were separated by SDS-PAGE using a 4–15% gradient gel (Bio-Rad, Hercules, CA) and transferred to PVDF membranes (Merck Millipore). Membranes were blocked with 5% BSA in TBST and incubated at 4°C with the following primary antibodies: anti-ERK (#9102), anti-phospho-ERK (#9101), anti-p38 (#9212), anti-phospho-p38 (#9211), and anti-*β*-actin (#4970) (all from Cell Signaling Technology, Danvers, MA). After washing, membranes were incubated with HRP-conjugated secondary antibodies (Cell Signaling Technology) and developed using enhanced chemiluminescence (Thermo Fisher Scientific). Images were captured using a ChemiDoc imaging system (Bio-Rad). Membranes were stripped using a commercial stripping buffer (Bionics, Korea) and reprobed with anti-*β*-actin antibody as a loading control. Band intensities were quantified by ImageJ (NIH, open source software).

### Single-cell RNA-seq

The publicly available single-cell RNA-sequencing dataset GSE247264 was analyzed, comprising small intestinal tissue samples from patients with Crohn’s disease and healthy controls. High-quality cells were selected using the following criteria based on the unique molecular identifiers (UMI) count matrix for each sample: (1) expression of >500 genes, (2) <3,500 genes per cell, and (3) <15% mitochondrial gene content. Potential doublets were identified and removed using DoubletFinder (v2.0.3). Data preprocessing and integration were performed using the Seurat v4 workflow, including highly variable gene selection, principal component analysis (PCA), and unsupervised clustering. B cell populations were identified based on canonical markers (CD19 and CD20), and corresponding clusters were extracted for further analysis. Sub-clustering of Bm cells was conducted using the same analytical pipeline to resolve distinct functional subsets within this compartment.

### Spatial transcriptomic using 10x Xenium

Spatial transcriptomic analysis was performed using the 10x Genomics Xenium platform. OCT-embedded frozen tissue blocks from inflamed regions of the small intestine were obtained from two patients with Crohn’s disease. Sections were mounted onto Xenium slides and processed according to the manufacturer’s protocol, including probe hybridization, ligation, and signal amplification. Autofluorescence quenching and nuclear staining were followed by imaging and spatial transcriptomic profiling using the Xenium Analyzer (XOA v1.7.6). Initial processing was conducted using XeniumRanger (v1.7.1), and cells with fewer than 10 detected genes were excluded. Downstream analysis was performed using standard workflows in Seurat v4.

### Statistical analysis

All statistical analyzes were performed using GraphPad Prism (GraphPad, La Jolla, CA). Comparisons between two groups were conducted using a two-tailed unpaired Student’s *t*-test. For comparisons involving more than two groups, either one-way ANOVA followed by Tukey’s multiple comparisons test or two-way ANOVA with Bonferroni’s post-hoc test was used, as appropriate. All data are mean ± SD. Statistical significance was defined as *p < 0.05, **p < 0.01, or ***p < 0.001.

## Supplementary Material

Supplementary Figures for 2nd revision.pdf Supplementary Figures for 2nd revision.pdf

## Data Availability

All sequencing data generated in this study are publicly available in the NCBI databases. Whole-genome sequencing data have been deposited in the BioProject under accession number PRJNA1175450. The spatial transcriptomic dataset generated using the 10x Genomics Xenium platform has been deposited under accession number GSE306881. In addition, we analyzed a publicly available single-cell RNA sequencing dataset with the accession number GSE247264. All other data supporting the findings of this study are available within the article and its supplementary information files.

## References

[1] Belkaid Y, Harrison OJ. Homeostatic immunity and the microbiota. Immunity. 2017;46:562–576. doi: 10.1016/j.immuni.2017.04.008. 28423337 PMC5604871

[2] Kamada N, Seo SU, Chen GY, Nunez G. Role of the gut microbiota in immunity and inflammatory disease. Nat Rev Immunol. 2013;13:321–335. doi: 10.1038/nri3430. 23618829

[3] O'Neill I, Schofield Z, Hall LJ. Exploring the role of the microbiota member *Bifidobacterium* in modulating immune-linked diseases. Emerg Top Life Sci. 2017;1:333–349. doi: 10.1042/ETLS20170058. 33525778 PMC7288987

[4] Li C, Peng K, Xiao S, Long Y, Yu Q. The role of *Lactobacillus* in inflammatory bowel disease: from actualities to prospects. Cell Death Discov. 2023;9:361. doi: 10.1038/s41420-023-01666-w. 37773196 PMC10541886

[5] Bender MJ, McPherson AC, Phelps CM, Pandey SP, Laughlin CR, Shapira JH, Medina Sanchez L, Rana M, Richie TG, Mims TS, et al. Dietary tryptophan metabolite released by intratumoral *Lactobacillus reuteri* facilitates immune checkpoint inhibitor treatment. Cell. 2023;186:1846–1862. doi: 10.1016/j.cell.2023.03.011. 37028428 PMC10148916

[6] Kim S, Lee S, Kim TY, Lee SH, Seo SU, Kweon MN. Newly isolated *Lactobacillus paracasei* strain modulates lung immunity and improves the capacity to cope with influenza virus infection. Microbiome. 2023;11:260. doi: 10.1186/s40168-023-01687-8. 37996951 PMC10666316

[7] Furusawa Y, Obata Y, Fukuda S, Endo TA, Nakato G, Takahashi D, Nakanishi Y, Uetake C, Kato K, Murakami S, et al. Commensal microbe-derived butyrate induces the differentiation of colonic regulatory T cells. Natur. 2013;504:446–450. doi: 10.1038/nature12721. 24226770

[8] Wlodarska M, Luo C, Kolde R, d'Hennezel E, Annand JW, Heim CE, d’Hennezel E, Krastel P, Schmitt EK, Omar AS, et al. Indoleacrylic acid produced by commensal Peptostreptococcus species suppresses inflammation. Cell Host Microbe. 2017;22:25–37. doi: 10.1016/j.chom.2017.06.007. 28704649 PMC5672633

[9] Zelante T, Iannitti RG, Cunha C, De Luca A, Giovannini G, Pieraccini G, Zecchi R, D’Angelo C, Massi-Benedetti C, Fallarino F, et al. Tryptophan catabolites from microbiota engage aryl hydrocarbon receptor and balance mucosal reactivity via interleukin-22. Immunity. 2013;39:372–385. doi: 10.1016/j.immuni.2013.08.003. 23973224

[10] Macho Fernandez E, Valenti V, Rockel C, Hermann C, Pot B, Boneca IG, Grangette C. Anti-inflammatory capacity of selected *lactobacilli* in experimental colitis is driven by NOD2-mediated recognition of a specific peptidoglycan-derived muropeptide. Gut. 2011;60:1050–1059. doi: 10.1136/gut.2010.232918. 21471573

[11] Wu Z, Pan D, Guo Y, Sun Y, Zeng X. Peptidoglycan diversity and anti-inflammatory capacity in *Lactobacillus* strains. Carbohydr Polym. 2015;128:130–137. doi: 10.1016/j.carbpol.2015.04.026. 26005148

[12] Grigorian A, Araujo L, Naidu NN, Place DJ, Choudhury B, Demetriou M. N-acetylglucosamine inhibits Th1/Th17 cell responses and treats experimental autoimmune encephalomyelitis. J Biol Chem. 2011;286:40133–40141. doi: 10.1074/jbc.M111.277814. 21965673 PMC3220534

[13] Gao J, Zhao X, Hu S, Huang Z, Hu M, Jin S, Lu B, Sun K, Wang Z, Fu J, et al. Gut microbial DL-endopeptidase alleviates Crohn's disease via the NOD2 pathway. Cell Host Microbe. 2022;30:1435–1449. doi: 10.1016/j.chom.2022.08.002. 36049483

[14] Saraiva M, O'Garra A. The regulation of IL-10 production by immune cells. Nat Rev Immunol. 2010;10:170–181. doi: 10.1038/nri2711. 20154735

[15] Uhlig HH, Coombes J, Mottet C, Izcue A, Thompson C, Fanger A, Tannapfel A, Fontenot JD, Ramsdell F, Powrie F. Characterization of Foxp3^+^CD4^+^CD25^+^ and IL-10-secreting CD4^+^CD25^+^ T cells during cure of colitis. J Immunol. 2006;177:5852–5860. doi: 10.4049/jimmunol.177.9.5852. 17056509 PMC6108413

[16] Barnes MJ, Powrie F. Regulatory T cells reinforce intestinal homeostasis. Immunity. 2009;31:401–411. doi: 10.1016/j.immuni.2009.08.011. 19766083

[17] Hayashi A, Sato T, Kamada N, Mikami Y, Matsuoka K, Hisamatsu T, Hibi T, Roers A, Yagita H, Ohteki T, et al. A single strain of *Clostridium butyricum* induces intestinal IL-10-producing macrophages to suppress acute experimental colitis in mice. Cell Host Microbe. 2013;13:711–722. doi: 10.1016/j.chom.2013.05.013. 23768495

[18] Round JL, Mazmanian SK. Inducible Foxp3^+^ regulatory T-cell development by a commensal bacterium of the intestinal microbiota. Proc Natl Acad Sci USA. 2010;107:12204–12209. doi: 10.1073/pnas.0909122107. 20566854 PMC2901479

[19] Mishima Y, Oka A, Liu B, Herzog JW, Eun CS, Fan TJ, Bulik-Sullivan E, Carroll IM, Hansen JJ, Chen L, et al. Microbiota maintain colonic homeostasis by activating TLR2/MyD88/PI3K signaling in IL-10-producing regulatory B cells. J Clin Invest. 2019;129:3702–3716. doi: 10.1172/JCI93820. 31211700 PMC6715367

[20] Rosser EC, Mauri C. Regulatory B cells: origin, phenotype, and function. Immunity. 2015;42:607–612. doi: 10.1016/j.immuni.2015.04.005. 25902480

[21] Shimomura Y, Mizoguchi E, Sugimoto K, Kibe R, Benno Y, Mizoguchi A, Bhan AK. Regulatory role of B-1 B cells in chronic colitis. Int Immunol. 2008;20:729–737. doi: 10.1093/intimm/dxn031. 18375938

[22] Blair PA, Norena LY, Flores-Borja F, Rawlings DJ, Isenberg DA, Ehrenstein MR, Noreña LY, Mauri C. CD19^+^CD24^hi^CD38^hi^ B cells exhibit regulatory capacity in healthy individuals but are functionally impaired in systemic Lupus Erythematosus patients. Immunity. 2010;32:129–140. doi: 10.1016/j.immuni.2009.11.009. 20079667

[23] Fu Y, Wang Z, Yu B, Lin Y, Huang E, Liu R, Zhao C, Lu M, Xu W, Chu Y. Intestinal CD11b^+^ B cells ameliorate colitis by secreting Immunoglobulin A. Front Immunol. 2021;12:697725. doi: 10.3389/fimmu.2021.697725. 34804004 PMC8595478

[24] Wong JB, Hewitt SL, Heltemes-Harris LM, Mandal M, Johnson K, Rajewsky K, Koralov SB, Clark MR, Farrar MA, Skok JA. B-1a cells acquire their unique characteristics by bypassing the pre-BCR selection stage. Nat Commun. 2019;10:4768. doi: 10.1038/s41467-019-12824-z. 31628339 PMC6802180

[25] Ding Q, Yeung M, Camirand G, Zeng Q, Akiba H, Yagita H, Chalasani G, Sayegh MH, Najafian N, Rothstein DM. Regulatory B cells are identified by expression of TIM-1 and can be induced through TIM-1 ligation to promote tolerance in mice. J Clin Invest. 2011;121:3645–3656. doi: 10.1172/JCI46274. 21821911 PMC3163958

[26] Yanaba K, Bouaziz JD, Haas KM, Poe JC, Fujimoto M, Tedder TF. A regulatory B cell subset with a unique CD1d^hi^CD5^+^ phenotype controls T cell-dependent inflammatory responses. Immunity. 2008;28:639–650. doi: 10.1016/j.immuni.2008.03.017. 18482568

[27] Matsushita T, Yanaba K, Bouaziz JD, Fujimoto M, Tedder TF. Regulatory B cells inhibit EAE initiation in mice while other B cells promote disease progression. J Clin Invest. 2008:3420–3430. doi: 10.1172/JCI36030; 118:18802481 PMC2542851

[28] Mauri C, Gray D, Mushtaq N, Londei M. Prevention of arthritis by interleukin 10-producing B cells. J Exp Med. 2003;197:489–501. doi: 10.1084/jem.20021293. 12591906 PMC2193864

[29] Heufler C, Koch F, Stanzl U, Topar G, Wysocka M, Trinchieri G, Enk A, Steinman RM, Romani N, Schuler G. Interleukin-12 is produced by dendritic cells and mediates T helper 1 development as well as interferon-gamma production by T helper 1 cells. Eur J Immunol. 1996;26:659–668. doi: 10.1002/eji.1830260323. 8605935

[30] Verma R, Lee C, Jeun EJ, Yi J, Kim KS, Ghosh A, Byun S, Kang H, Jun C, Jan G, et al. Cell surface polysaccharides of Bifidobacterium bifidum induce the generation of Foxp3^+^ regulatory T cells. Sci Immunol. 2018;3. doi: 10.1126/sciimmunol.aat6975. 30341145

[31] Rubtsov YP, Rasmussen JP, Chi EY, Fontenot J, Castelli L, Ye X, Treuting P, Siewe L, Roers A, Henderson WR, et al. Regulatory T cell-derived interleukin-10 limits inflammation at environmental interfaces. Immunity. 2008;28:546–558. doi: 10.1016/j.immuni.2008.02.017. 18387831

[32] Jansen K, Cevhertas L, Ma S, Satitsuksanoa P, Akdis M, van de Veen W. Regulatory B cells, A to Z. Allergy. 2021;76:2699–2715. doi: 10.1111/all.14763. 33544905

[33] Aziz M, Holodick NE, Rothstein TL, Wang P. The role of B-1 cells in inflammation. Immunol Res. 2015;63:153–166. doi: 10.1007/s12026-015-8708-3. 26427372 PMC4651765

[34] Griffin DO, Rothstein TL. Human “orchestrator” CD11b^+^ B1 cells spontaneously secrete interleukin-10 and regulate T-cell activity. Mol Med. 2012;18:1003–1008. doi: 10.2119/molmed.2012.00203. 22634719 PMC3459484

[35] Verma R, Lee C, Jeun EJ, Yi J, Kim KS, Ghosh A, Byun S, Kang H, Jun C, Jan G, et al. Cell surface polysaccharides of *Bifidobacterium bifidum* induce the generation of Foxp3^+^ regulatory T cells. Sci Immunol. 2018;3:eaat6975. doi: 10.1126/sciimmunol.aat6975. 30341145

[36] Wolf AJ, Underhill DM. Peptidoglycan recognition by the innate immune system. Nat Rev Immunol. 2018;18:243–254. doi: 10.1038/nri.2017.136. 29292393

[37] Green DW. The bacterial cell wall as a source of antibacterial targets. Expert Opin Ther Targets. 2002;6:1–19. doi: 10.1517/14728222.6.1.1. 11901475

[38] Lee SH, Cho SY, Yoon Y, Park C, Sohn J, Jeong JJ, Jeon B, Jang M, An C, Kim YY, et al. *Bifidobacterium bifidum* strains synergize with immune checkpoint inhibitors to reduce tumour burden in mice. Nat Microbiol. 2021;6:277–288. doi: 10.1038/s41564-020-00831-6. 33432149

[39] Medzhitov R. Toll-like receptors and innate immunity. Nat Rev Immunol. 2001;1:135–145. doi: 10.1038/35100529. 11905821

[40] Groft SG, Nagy N, Boom WH, Harding CV. Toll-like receptor 2-Tpl2-dependent ERK signaling drives inverse interleukin 12 regulation in dendritic cells and macrophages. Infect Immun. 2020;89:e00323. doi: 10.1128/IAI.00323-20. 33077627 PMC7927937

[41] Lucas M, Zhang X, Prasanna V, Mosser DM. ERK activation following macrophage FcgammaR ligation leads to chromatin modifications at the IL-10 locus. J Immunol. 2005;175:469–477. doi: 10.4049/jimmunol.175.1.469. 15972681

[42] Francisco S, Arranz A, Merino J, Punzon C, Perona R, Fresno M. Early p38 activation regulated by MKP-1 is determinant for high levels of IL-10 expression through TLR2 activation. Front Immunol. 2021;12:660065. doi: 10.3389/fimmu.2021.660065. 34234775 PMC8256158

[43] Brown EM, Kenny DJ, Xavier RJ. Gut microbiota regulation of T cells during inflammation and autoimmunity. Annu Rev Immunol. 2019;37:599–624. doi: 10.1146/annurev-immunol-042718-041841. 31026411

[44] Levast B, Li Z, Madrenas J. The role of IL-10 in microbiome-associated immune modulation and disease tolerance. Cytokine. 2015;75:291–301. doi: 10.1016/j.cyto.2014.11.027. 25542093

[45] Atarashi K, Tanoue T, Shima T, Imaoka A, Kuwahara T, Momose Y, Cheng G, Yamasaki S, Saito T, Ohba Y, et al. Induction of colonic regulatory T cells by indigenous *Clostridium* species. Sci. 2011;331:337–341. doi: 10.1126/science.1198469. PMC3969237 21205640

[46] Jeon SG, Kayama H, Ueda Y, Takahashi T, Asahara T, Tsuji H, Kiyono H, Ma JS, Kusu T, Okumura R, et al. Probiotic *Bifidobacterium breve* induces IL-10-producing Tr1 cells in the colon. PLoS Pathog. 2012;8:e1002714. doi: 10.1371/journal.ppat.1002714. 22693446 PMC3364948

[47] Zegarra Ruiz DF, Kim DV, Norwood K, Saldana-Morales FB, Kim M, Ng C, Callaghan R, Uddin M, Chang L, Longman RS, et al. Microbiota manipulation to increase macrophage IL-10 improves colitis and limits colitis-associated colorectal cancer. Gut Microbes. 2022;14:2119054. doi: 10.1080/19490976.2022.2119054. 36062329 PMC9450902

[48] Durant L, Stentz R, Noble A, Brooks J, Gicheva N, Reddi D, O’Connor MJ, Hoyles L, McCartney AL, Man R, et al. *Bacteroides thetaiotaomicron*-derived outer membrane vesicles promote regulatory dendritic cell responses in health but not in inflammatory bowel disease. Microbiome. 2020;8:88. doi: 10.1186/s40168-020-00868-z. 32513301 PMC7282036

[49] Yanaba K, Yoshizaki A, Asano Y, Kadono T, Tedder TF, Sato S. IL-10-producing regulatory B10 cells inhibit intestinal injury in a mouse model. Am J Pathol. 2011;178:735–743. doi: 10.1016/j.ajpath.2010.10.022. 21281806 PMC3069829

[50] Matsushita T, Horikawa M, Iwata Y, Tedder TF. Regulatory B cells (B10 cells) and regulatory T cells have independent roles in controlling experimental autoimmune encephalomyelitis initiation and late-phase immunopathogenesis. J Immunol. 2010;185:2240–2252. doi: 10.4049/jimmunol.1001307. 20624940 PMC3717968

[51] Fillatreau S, Sweenie CH, McGeachy MJ, Gray D, Anderton SM. B cells regulate autoimmunity by provision of IL-10. Nat Immunol. 2002;3:944–950. doi: 10.1038/ni833. 12244307

[52] Xiao S, Bod L, Pochet N, Kota SB, Hu D, Madi A, Kilpatrick J, Shi J, Ho A, Zhang H, et al. Checkpoint receptor TIGIT expressed on Tim-1^+^ B cells regulates tissue inflammation. Cell Rep. 2020;32:107892. doi: 10.1016/j.celrep.2020.107892. 32668241 PMC7496220

[53] Matsumura Y, Watanabe R, Fujimoto M. Suppressive mechanisms of regulatory B cells in mice and humans. Int Immunol. 2023;35:55–65. doi: 10.1093/intimm/dxac048. 36153768 PMC9918854

[54] Maseda D, Candando KM, Smith SH, Kalampokis I, Weaver CT, Plevy SE, Poe JC, Tedder TF. Peritoneal cavity regulatory B cells (B10 cells) modulate IFN-γ​​​​​​^+^CD4^+^ T cell numbers during colitis development in mice. J Immunol. 2013;191:2780–2795. doi: 10.4049/jimmunol.1300649. 23918988 PMC3770313

[55] Kaji R, Kiyoshima-Shibata J, Nagaoka M, Nanno M, Shida K. Bacterial teichoic acids reverse predominant IL-12 production induced by certain lactobacillus strains into predominant IL-10 production via TLR2-dependent ERK activation in macrophages. J Immunol. 2010;184:3505–3513. doi: 10.4049/jimmunol.0901569. 20190136

[56] Garcia-Vello P, Tytgat HLP, Elzinga J, Van Hul M, Plovier H, Tiemblo-Martin M, Cani PD, Nicolardi S, Fragai M, De Castro C, et al. The lipooligosaccharide of the gut symbiont *Akkermansia muciniphila* exhibits a remarkable structure and TLR signaling capacity. Nat Commun. 2024;15:8411. doi: 10.1038/s41467-024-52683-x. 39333588 PMC11436972

[57] Ramakrishna C, Kujawski M, Chu H, Li L, Mazmanian SK, Cantin EM. *Bacteroides fragilis* polysaccharide A induces IL-10 secreting B and T cells that prevent viral encephalitis. Nat Commun. 2019;10:2153. doi: 10.1038/s41467-019-09884-6. 31089128 PMC6517419

[58] Kwan JMC, Liang Y, Ng EWL, Sviriaeva E, Li C, Zhao Y, Zhang X, Liu X, Wong SH, Qiao Y. In silico MS/MS prediction for peptidoglycan profiling uncovers novel anti-inflammatory peptidoglycan fragments of the gut microbiota. Chem Sci. 2024;15:1846–1859. doi: 10.1039/D3SC05819K. 38303944 PMC10829024

[59] Torrens G, Cava F. Mechanisms conferring bacterial cell wall variability and adaptivity. Biochem Soc Trans. 2024;52:1981–1993. doi: 10.1042/BST20230027. 39324635 PMC11555704

[60] Wheeler R, Gomperts Boneca I. The hidden base of the iceberg: gut peptidoglycome dynamics is foundational to its influence on the host. Gut Microbes. 2024;16:2395099. doi: 10.1080/19490976.2024.2395099. 39239828 PMC11382707

[61] Murakami M, Tsubata T, Shinkura R, Nisitani S, Okamoto M, Yoshioka H, Usui T, Miyawaki S, Honjo T. Oral administration of lipopolysaccharides activates B-1 cells in the peritoneal cavity and lamina propria of the gut and induces autoimmune symptoms in an autoantibody transgenic mouse. J Exp Med. 1994;180:111–121. doi: 10.1084/jem.180.1.111. 8006578 PMC2191544

[62] Liu T, Ren J, Wang W, Wei XW, Shen GB, Liu YT, Luo M, Xu G, Shao B, Deng S, et al. Treatment of dextran sodium sulfate-induced experimental colitis by adoptive transfer of peritoneal cells. Sci Rep. 2015;5:16760. doi: 10.1038/srep16760. 26565726 PMC4643275

[63] Simon Q, Pers JO, Cornec D, Le Pottier L, Mageed RA, Hillion S. In-depth characterization of CD24^hi^CD38^hi^ transitional human B cells reveals different regulatory profiles. J Allergy Clin Immunol. 2016;137:1577–1584. doi: 10.1016/j.jaci.2015.09.014. 26525227

[64] Iwata Y, Matsushita T, Horikawa M, Dilillo DJ, Yanaba K, Venturi GM, Szabolcs PM, Bernstein SH, Magro CM, Williams AD, et al. Characterization of a rare IL-10-competent B-cell subset in humans that parallels mouse regulatory B10 cells. Blood. 2011;117:530–541. doi: 10.1182/blood-2010-07-294249. 20962324 PMC3031478

[65] Sidira M, Saxami G, Dimitrellou D, Santarmaki V, Galanis A, Kourkoutas Y. Monitoring survival of *Lactobacillus casei* ATCC 393 in probiotic yogurts using an efficient molecular tool. J Dairy Sci. 2013;96:3369–3377. doi: 10.3168/jds.2012-6343. 23498002

[66] Hidalgo-Cantabrana C, Algieri F, Rodriguez-Nogales A, Vezza T, Martinez-Camblor P, Margolles A, Martínez-Camblor P, Ruas-Madiedo P, Gálvez J. Effect of a ropy exopolysaccharide-producing *Bifidobacterium* animalis subsp. lactis strain orally administered on DSS-induced colitis mice model. Front Microbiol. 2016;7:868. doi: 10.3389/fmicb.2016.00868. 27375589 PMC4900019

[67] Masuko T, Minami A, Iwasaki N, Majima T, Nishimura S, Lee YC. Carbohydrate analysis by a phenol-sulfuric acid method in microplate format. Anal Biochem. 2005;339:69–72. doi: 10.1016/j.ab.2004.12.001. 15766712

[68] Rosenthal RS, Dziarski R. Isolation of peptidoglycan and soluble peptidoglycan fragments. Methods Enzymol. 1994;235:253–285. 8057899 10.1016/0076-6879(94)35146-5

[69] Kuhner D, Stahl M, Demircioglu DD, Bertsche U. From cells to muropeptide structures in 24 h: peptidoglycan mapping by UPLC-MS. Sci Rep. 2014;4:7494. doi: 10.1038/srep07494. 25510564 PMC4267204

[70] Livak KJ, Schmittgen TD. Analysis of relative gene expression data using real-time quantitative PCR and the 2^-Delta Delta C(T)^ method. Methods. 2001;25:402–408. doi: 10.1006/meth.2001.1262. 11846609

[71] Sheu SJ, Hwang WZ, Chen HC, Chiang YC, Tsen HY. Development and use of tuf gene-based primers for the multiplex PCR detection of *Lactobacillus acidophilus*, *Lactobacillus casei* group, *Lactobacillus delbrueckii*, and *Bifidobacterium longum* in commercial dairy products. J Food Prot. 2009;72:93–100. doi: 10.4315/0362-028X-72.1.93. 19205469

[72] HaileMariam M, Eguez RV, Singh H, Bekele S, Ameni G, Pieper R, Yu Y. S-Trap, an ultrafast sample-preparation approach for shotgun proteomics. J Proteome Res. 2018;17:2917–2924. doi: 10.1021/acs.jproteome.8b00505. 30114372

[73] Ahn HS, Kim JH, Jeong H, Yu J, Yeom J, Song SH, Kim SS, Kim IJ, Kim K. Differential urinary proteome analysis for predicting prognosis in type 2 diabetes patients with and without renal dysfunction. Int J Mol Sci. 2020;21(12):4236. doi: 10.3390/ijms21124236. 32545899 PMC7352871

