# Supplementary material for: Peptidoglycan from Bifidobacterium adolescentis enhances IL-10 production in regulatory B cells to alleviate gut inflammation
Source: Gut Microbes. 2026 Jan 9;18(1):2611603. doi: 10.1080/19490976.2025.2611603 (PMC12795277; doi:10.1080/19490976.2025.2611603)
Supplement: Supplementary Figures for 2nd revision.pdf [file KGMI_A_2611603_SM3026.pdf]

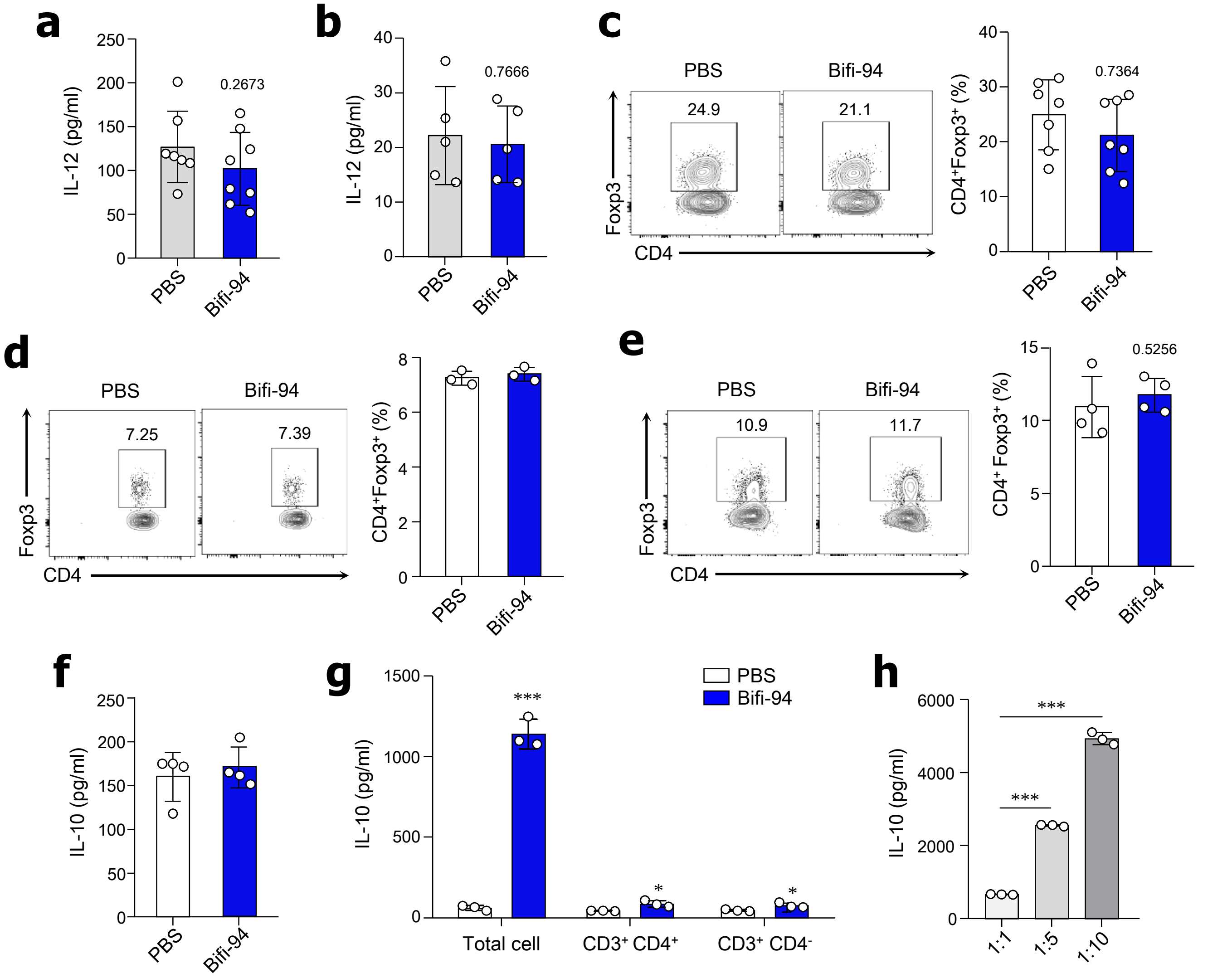

Figure S1. Oral administration of *B. adolescentis* Bifi-94 enhances IL-10 production but does not affect CD4$^+$Foxp3$^+$ T cell frequencies

(**a–d**) B6 mice were orally administered *B. adolescentis* Bifi-94 daily for 2 weeks. IL-12 levels were measured in (**a**) colon tissues (n = 7–8) and (**b**) spleen homogenates (n = 5). Frequencies of CD4$^+$Foxp3$^+$ Treg cells were analyzed by flow cytometry in the (**c**) colon (n = 7) and (**d**) spleen (n = 3). (**e–f**) Naïve CD4$^+$ T cells isolated from the spleens of B6 mice were cultured under Treg-polarizing conditions in the presence or absence of Bifi-94 for 72 hours (n = 4). (**e**) Frequency of CD4$^+$Foxp3$^+$ Treg cells and (**f**) IL-10 levels in culture supernatants were measured by ELISA. (**g**) Splenocytes were sorted into CD3$^+$CD4$^+$ and CD3$^+$CD4$^-$ populations and co-cultured with live Bifi-94 for 72 hours. IL-10 production was quantified by ELISA in both populations (n = 3). (**h**) PEC cells were co-cultured with *B. adolescentis* Bifi-94 at the indicated cell-to-bacteria ratios for 72 hours, and IL-10 levels in the supernatants were measured by ELISA (n = 3). Data are presented as mean ± SD. Statistical analysis was performed using a two-tailed paired Student's t-test or one-way ANOVA followed by Tukey's post-hoc test, as appropriate. $*p < 0.05$, and $***p < 0.001$ were considered statistically significant.

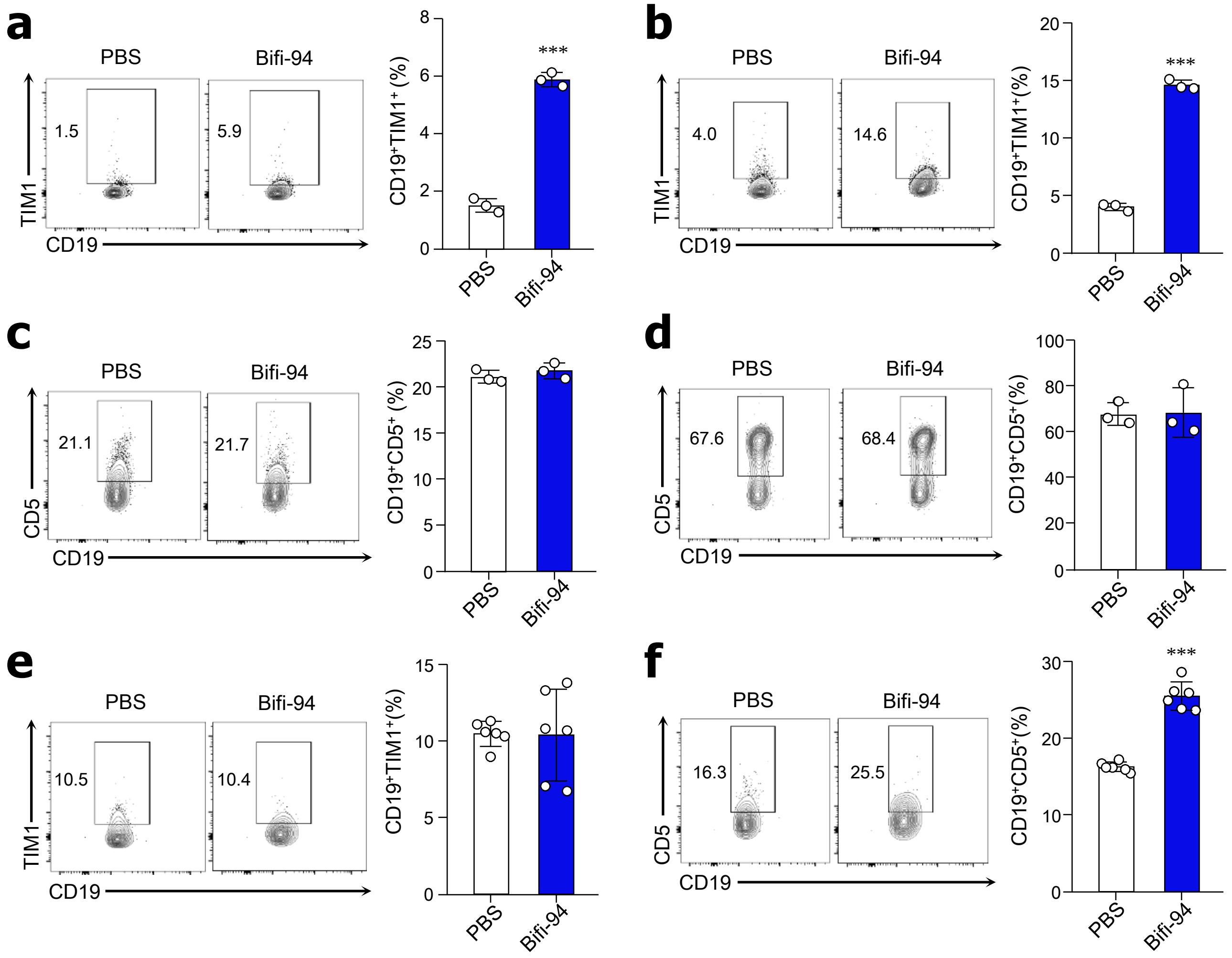

Figure S2. *B. adolescentis* Bifi-94 induces differentiation of Breg cells

Mononuclear cells isolated from the spleen, colon, and PEC of B6 mice were incubated with Bifi-94 for 72 hours and analyzed by flow cytometry. Frequency of $CD19^{+}TIM\text{-}1^{+}$ Breg cells in the (**a**) spleen and (**b**) PEC (n = 3). Frequency of $CD19^{+}CD5^{+}$ cells in the (**c**) spleen and (**d**) PEC (n = 3). Frequency of (**e**) $CD19^{+}TIM\text{-}1^{+}$ cells and (**f**) $CD19^{+}CD5^{+}$ Breg cells in the colon (n = 6). Data are presented as mean ± SD and were combined from ≥2 independent experiments. Statistical analysis was performed using a two-tailed paired Student's t-test. ****p < 0.001* were considered statistically significant.

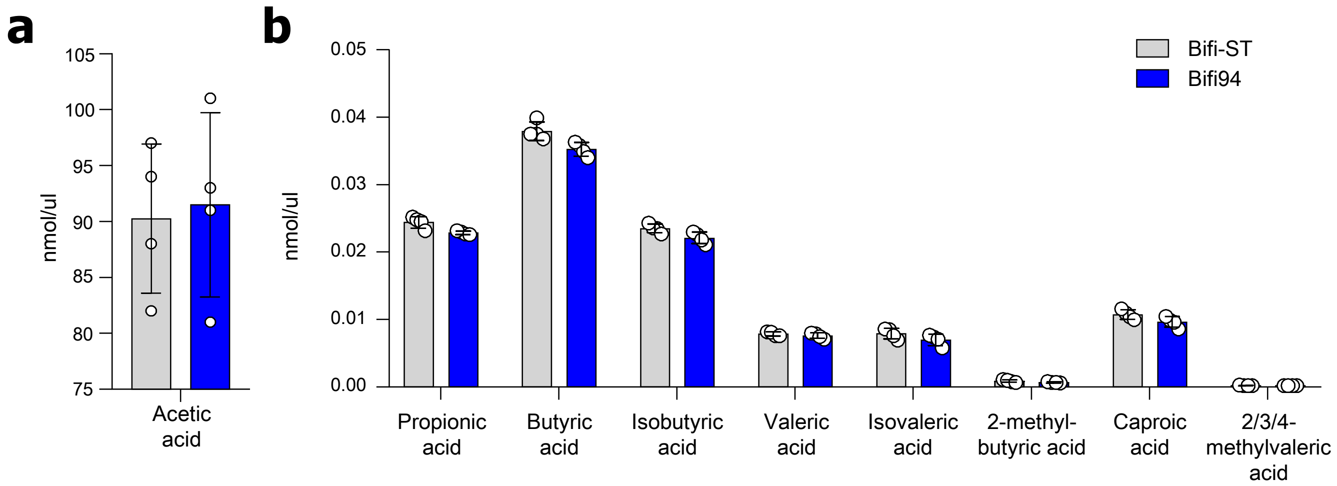

Figure S3. Quantification of short-chain fatty acids (SCFA) in culture supernatants

SCFA levels were measured in culture supernatants of *B. adolescentis* Bifi-ST and Bifi-94 strains. (**a**) Quantification of acetic acid (n = 3). (**b**) Quantification of other SCFA, including propionic and butyric acids (n = 3). Data are presented as mean ± SD. Statistical analysis was performed using a two-tailed paired Student's t-test.

(Lee S. *et al.* Figure S4)

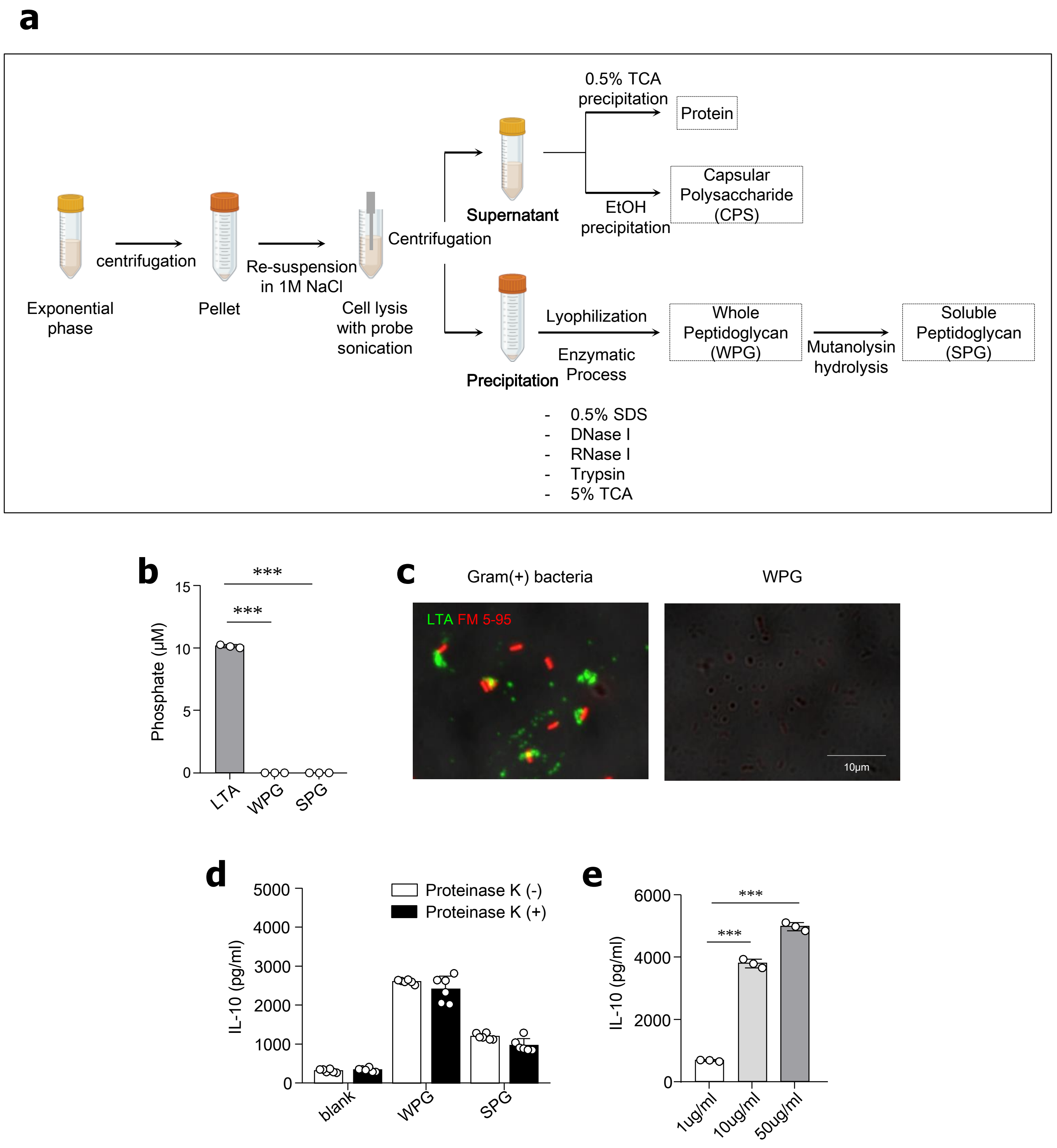

Figure S4. Workflow for purification of *B. adolescentis* Bifi-94 peptidoglycan (PG) and assessment of purity.

(a) Schematic overview of the purification procedure. Exponential-phase bacterial cells were lysed by probe sonication and sequentially treated with 1 M NaCl, 0.5% SDS, RNase, DNase, trypsin, and 5% TCA, followed by acetone and ethanol precipitation to remove membrane-associated materials, nucleic acids, proteins, lipoproteins, and teichoic acids. The resulting cell wall fraction was lyophilized to obtain purified whole PG (WPG), and soluble PG (SPG) was generated by mutanolysin digestion. **(b)** Teichoic acid contamination was assessed using an inorganic phosphate assay. Both WPG and SPG preparations showed negligible phosphate levels compared with an LTA positive control, confirming effective removal of teichoic acid-associated components. (**c**) Confocal images of Gram-positive bacteria (Bifi-94) and Bifi-94-derived WPG stained with FM 5-95 (red) and LTA (green). Samples were adhered to silane coated glass slides and permeabilized with lysozyme (1 mg/mL, 10 min, 37℃). Immunostaining was performed using anti-LTA IgG antibody (1:50, clone 55, Hycult Biotech, Uden, Netherlands) overnight, followed by Alexa Fluor 488-conjugated goat anti-mouse IgG (1:500, Invitrogen). Membranes were counterstained with FM 5-95 (2 μg/mL, Invitrogen). Images were acquired using an LSM 710 confocal microscope (Carl Zeiss). Scale bar = 10 μm. (**d**) To evaluate potential contamination by proteinaceous or lipoprotein components, peritoneal cavity (PEC) cells were stimulated for 72 hours with WPG (10 μg/mL) or SPG (10 μg/mL) from Bifi-94 that had been pre-treated with proteinase K (100 μg/mL), and IL-10 levels in the culture supernatants were measured by ELISA. IL-10 production remained unchanged following Proteinase K treatment, indicating minimal contribution of protein/lipoprotein contaminants to the immunostimulatory activity of the PG preparations. (**e**) PEC cells were co-cultured with the indicated concentrations of WPG derived from Bifi-94 for 72 hours, and IL-10 levels in the culture supernatants were measured by ELISA. Data are presented as mean ± SD. Statistical analyses were performed using paired two-tailed Student's t-tests or one-way ANOVA with Tukey's post-hoc test. ***$p < 0.001$ was considered statistically significant.

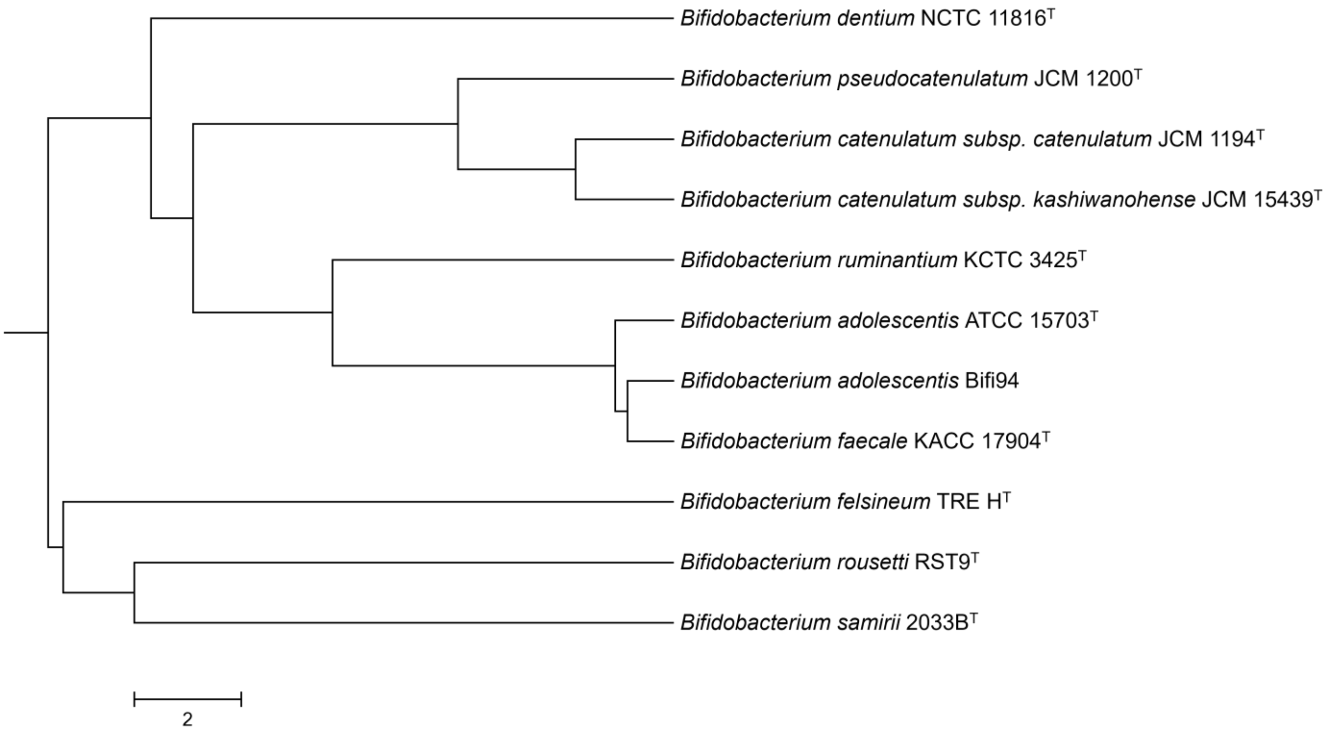

Figure S5. Phylogenetic relationship of *B. adolescentis* Bifi-94 and related taxa

A phylogenetic tree was constructed based on 16S rDNA sequences to determine the taxonomic relationship between *B. adolescentis* Bifi-94 and related bacterial strains. The tree illustrates the close phylogenetic proximity of Bifi-94 to the type strain and other members of the *B. adolescentis* clade.

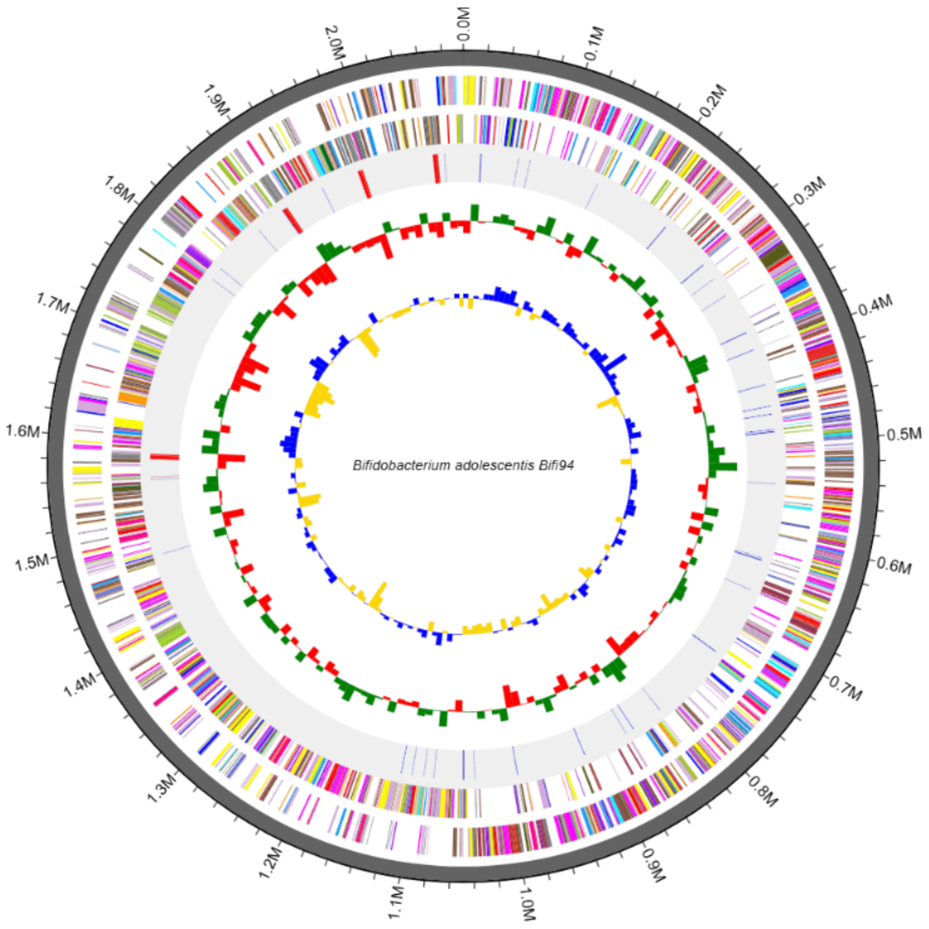

Information storage and processing

- A RNA processing and modification
- B Chromatin structure and dynamics
- J Translation, ribosomal structure and biogenesis
- K Transcription
- L Replication, recombination and repair

Cellular processes and signaling

- D Cell cycle control, cell division, chromosome partitioning
- M Cell wall/membrane/envelope biogenesis
- N Cell motility
- O Post-translational modification, protein turnover, and chaperones
- T Signal transduction mechanisms
- U Intracellular trafficking, secretion, and vesicular transport
- V Defense mechanisms
- W Extracellular structures
- Y Nuclear structure
- Z Cytoskeleton

Metabolism

- C Energy production and conversion
- G Carbohydrate transport and metabolism
- E Amino acid transport and metabolism
- F Nucleotide transport and metabolism
- H Coenzyme transport and metabolism
- I Lipid transport and metabolism
- P Inorganic ion transport and metabolism
- Q Secondary metabolites biosynthesis, transport and catabolism
- R General function prediction
- S Function unknown

Figure S6. Complete genome map of *B. adolescentis* Bifi-94 strain

Circular genome map of the *B. adolescentis* Bifi-94 strain. All coding sequences (CDSs) are categorized and color-coded according to COGs functional categories. The map illustrates the genomic organization and functional classification of annotated genes.

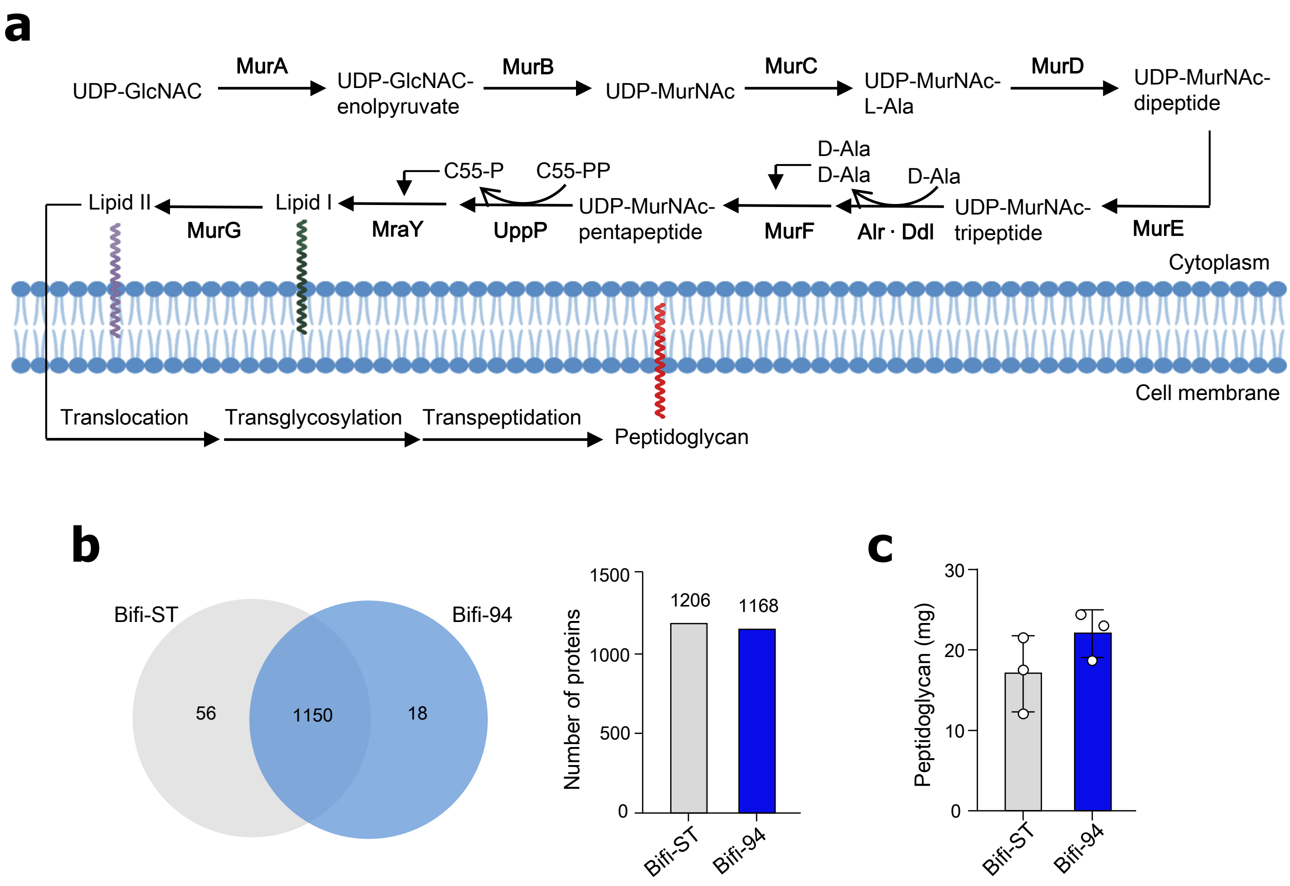

Figure S7. Comparison of PG biosynthesis pathways and protein profiles between Bifi-ST and Bifi-94 strains

(**a**) Schematic diagram of the bacterial PG biosynthesis pathway, highlighting key enzymes involved. MurA: UDP-N-acetylglucosamine 1-carboxyvinyltransferase; MurB: UDP-N-acetylmuramate dehydrogenase; MurC: UDP-N-acetylmuramate--L-alanine ligase; MurD: UDP-N-acetylmuramoyl-L-alanine--D-glutamate ligase; MurE: UDP-N-acetylmuramoyl-L-alanyl-D-glutamate--2,6-diaminopimelate ligase; MurF: UDP-N-acetylmuramoyl-tripeptide--D-alanyl-D-alanine ligase; MurG: Undecaprenyldiphospho-muramoylpentapeptide beta-N-acetylglucosaminyltransferase; MraY: Phospho-N-acetylmuramoyl-pentapeptide-transferase; UppP: Undecaprenyl-diphosphate phosphatase; Alr: Alanine racemase; Ddl: D-alanine--D-alanine ligase. (**b**) Venn diagrams illustrating the number identified by proteomic analysis in Bifi-ST and Bifi-94 strains. (**c**) Quantification of the total weight of PGN isolated from Bifi-ST and Bifi-94 strains (n = 3). Data are presented as mean ± SD. Statistical analysis was performed using a two-tailed paired Student’s t-test.

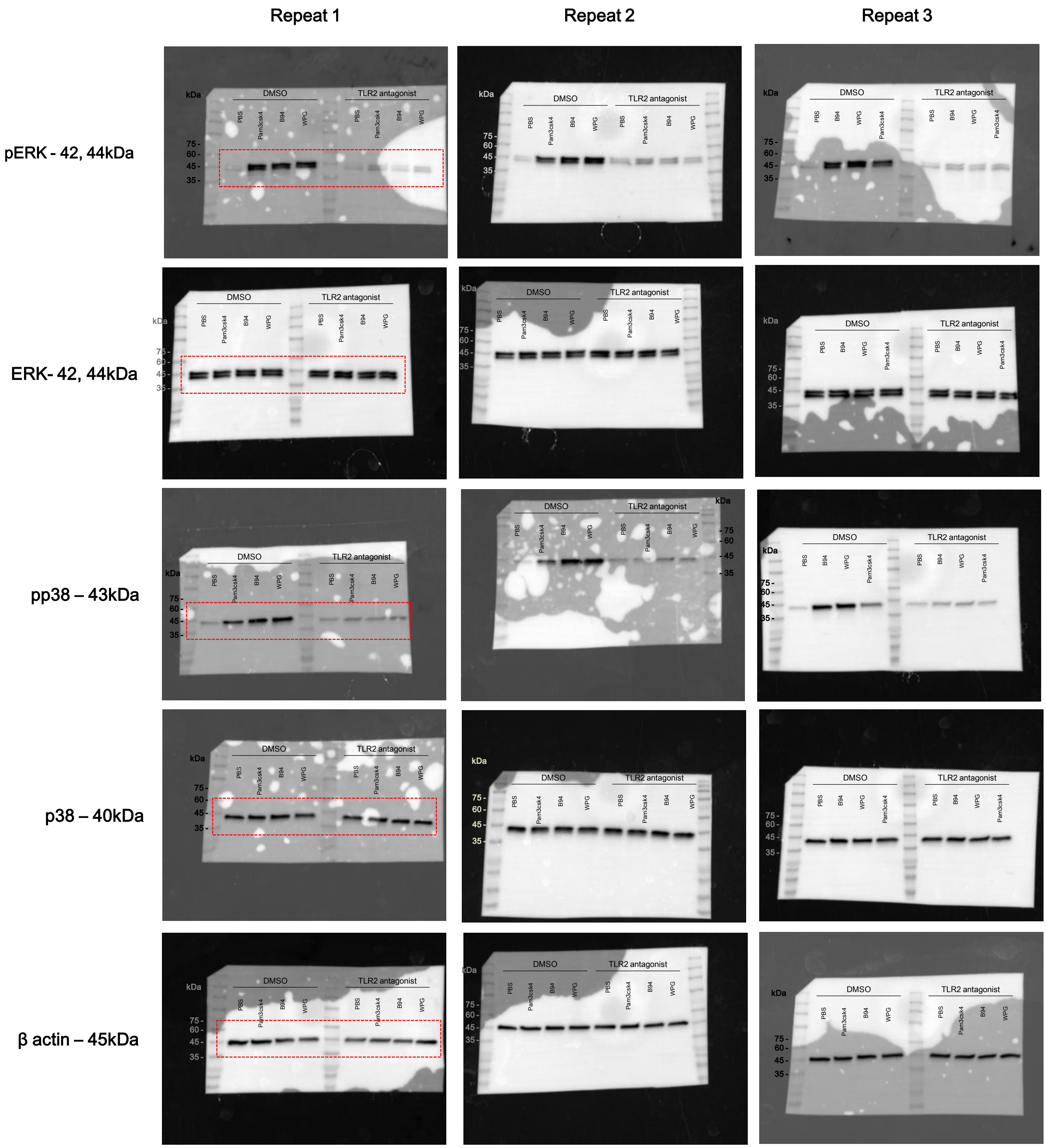

Figure S8. Uncropped and unprocessed Western blot images

Full-length Western blot images corresponding to the blots shown in Figure 6c. Red boxes indicate the regions that were cropped and presented in the main figure. All blots were processed under identical experimental conditions.

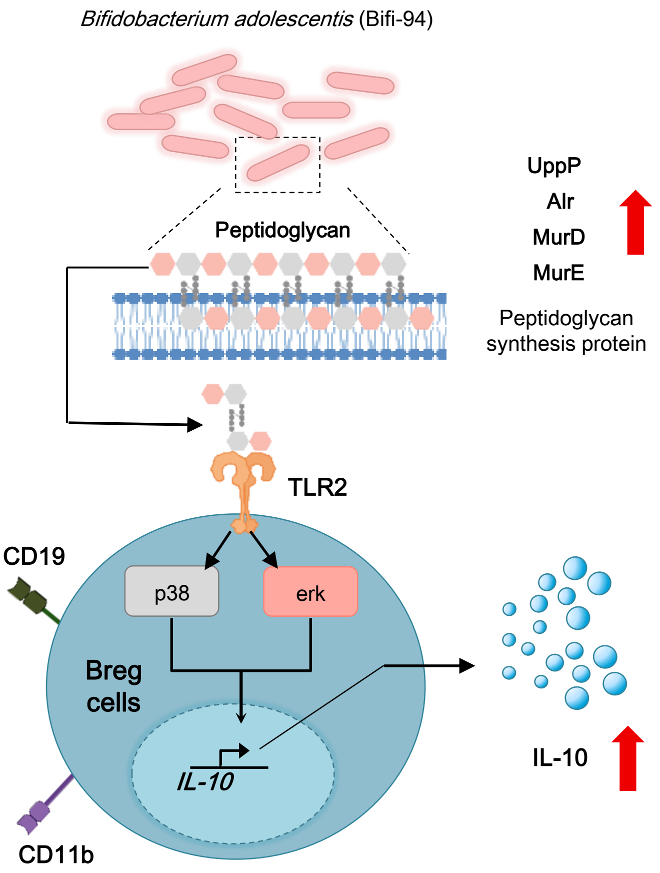

Figure S9. Schematic summary of IL-10 induction by Bifi-94 stimuli

Graphical representation of the proposed mechanism by which Bifi-94 and its WPG activate TLR2 signaling in $CD11b^{+}CD19^{+}$ Breg cells. Engagement of TLR2 triggers activation of the ERK and p38 pathways, leading to IL-10 production and suppression of intestinal inflammation.

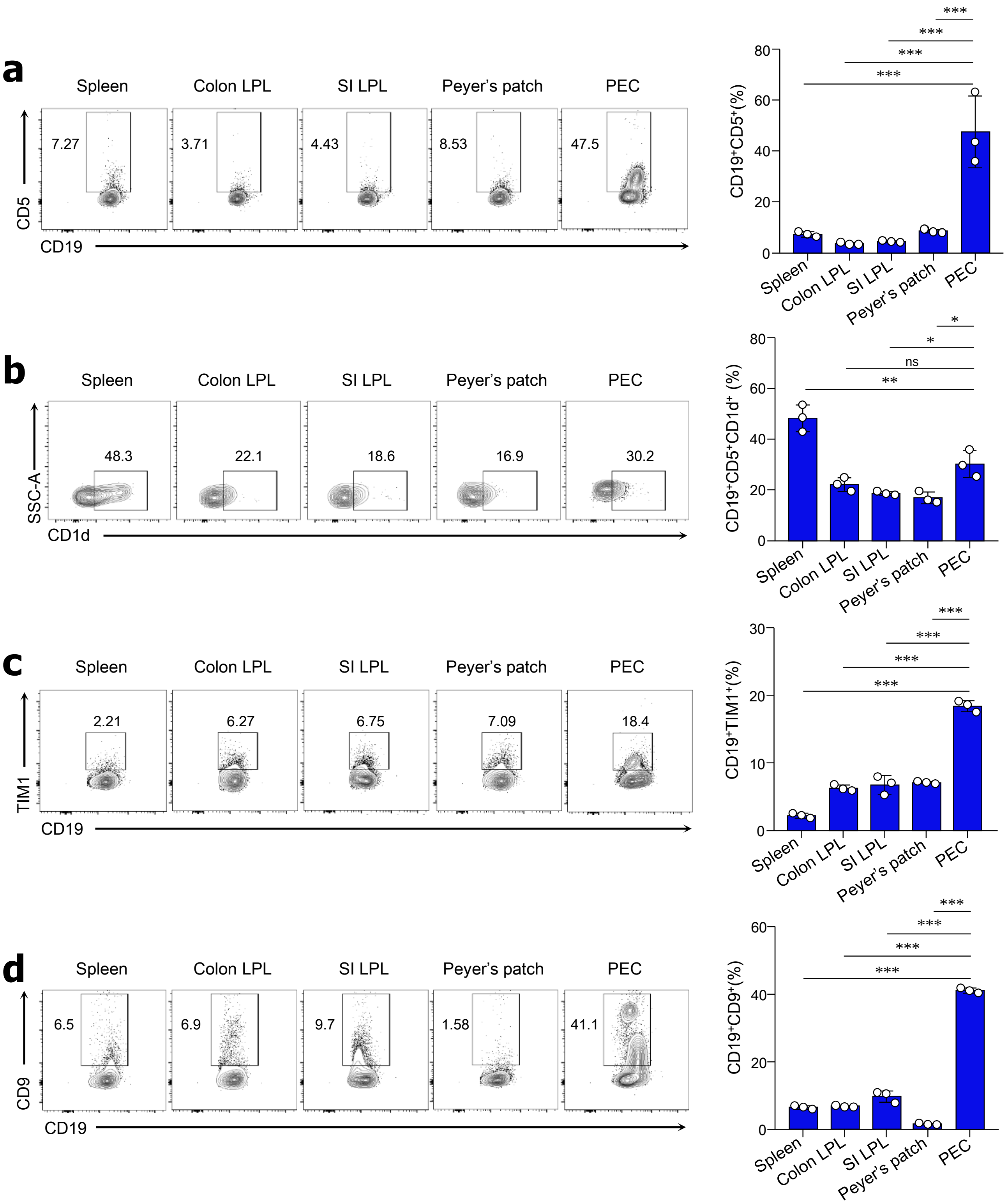

Figure S10. Breg cells are predominantly localized in the peritoneal cavity
(**a**–**d**) Mononuclear cells isolated from the spleen, colon, small intestine, Peyer's patch, and PEC of B6 mice and analyzed by flow cytometry (n = 3). (**a**) Frequency of $CD19^{+}CD5^{+}$ cells. (**b**) Frequency of $CD19^{+}CD5^{+}CD1d^{+}$ cells. (**c**) Frequency of $CD19^{+}TIM\text{-}1^{+}$ cells. (**d**) Frequency of $CD19^{+}CD9^{+}$ cells. Data are presented as mean ± SD. Statistical analysis was performed using one-way ANOVA followed by Tukey's post-hoc test. $^{*}p < 0.05$, $^{**}p < 0.01$ and $^{***}p < 0.001$ were considered statistically significant.

Table S1. Summary of genome annotation of Bifi-ST (KCTC 3216) and Bifi94 strains.

Strain	Base (bp)	No. of CDS*	GC contents (%)	No. of tRNA	No. of rRNA	ANIb† (%)
Bifi-ST	2,089,645	1,681	59.2	54	16	-
Bifi-94	2,097,731	1,666	59.4	54	13	97.92

* Coding sequences

† Average nucleotide identity based BLAST to type strain KCTC 3216

Table S2. Clinical characteristics.

	Sex	Age	Disease	
1	F	47	Colorectal cancer	Lamina propria lymphocyte preparation
2	M	27	Crohn’s disease	Lamina propria lymphocyte preparation
3	M	33	Colorectal cancer	Lamina propria lymphocyte preparation
4	F	38	Colorectal cancer	Lamina propria lymphocyte preparation
5	M	64	Colorectal cancer	Lamina propria lymphocyte preparation
6	F	58	Colorectal cancer	Lamina propria lymphocyte preparation
7	F	35	Colorectal cancer	Lamina propria lymphocyte preparation
8	M	44	Crohn’s disease	Lamina propria lymphocyte preparation
9	M	63	Colorectal cancer	Lamina propria lymphocyte preparation
10	F	32	Colorectal polyp	Lamina propria lymphocyte preparation
11	M	54	Colorectal cancer	Lamina propria lymphocyte preparation
12	M	27	Crohn’s disease	Lamina propria lymphocyte preparation
13	F	52	Colorectal cancer	Lamina propria lymphocyte preparation
14	M	35	Crohn’s disease	Spatial transcriptomics
15	M	18	Crohn’s disease	Spatial transcriptomics
